# DNA methylation clocks for dogs and humans

**DOI:** 10.1073/pnas.2120887119

**Published:** 2022-05-17

**Authors:** Steve Horvath, Ake T. Lu, Amin Haghani, Joseph A. Zoller, Caesar Z. Li, Andrea R. Lim, Robert T. Brooke, Ken Raj, Aitor Serres-Armero, Dayna L. Dreger, Andrew N. Hogan, Jocelyn Plassais, Elaine A. Ostrander

**Affiliations:** ^a^Department of Human Genetics, David Geffen School of Medicine, University of California, Los Angeles, CA 90095;; ^b^Department of Biostatistics, Fielding School of Public Health, University of California, Los Angeles, CA 90095;; ^c^Epigenetic Clock Development Foundation, Los Angeles, CA 90502;; ^d^Radiation Effects Department, Centre for Radiation, Chemical and Environmental Hazards, Public Health England, Chilton, Didcot OX11 0RQ, United Kingdom;; ^e^National Human Genome Research Institute, NIH, Bethesda, MD 20892

**Keywords:** dog, *Canis familiaris*, methylation, epigenetic clock, aging

## Abstract

DNA methylation profiles have been used to develop biomarkers of aging known as epigenetic clocks, which predict chronological age with remarkable accuracy and show promise for inferring health status as an indicator of biological age. Epigenetic clocks were first built to monitor human aging, but their underlying principles appear to be evolutionarily conserved, as they have now been successfully developed for many mammalian species. Here, we describe reliable and highly accurate epigenetic clocks shown to apply to 93 domestic dog breeds. The methylation profiles were generated using the mammalian methylation array, which utilizes DNA sequences that are conserved across all mammalian species. Canine epigenetic clocks were constructed to estimate age and also average time to death. We also present two highly accurate human–dog dual species epigenetic clocks (*R* = 0.97), which may facilitate the ready translation from canine to human use (or vice versa) of antiaging treatments being developed for longevity and preventive medicine. Finally, epigenome-wide association studies here reveal individual methylation sites that may underlie the inverse relationship between breed weight and lifespan. Overall, we describe robust biomarkers to measure aging and, potentially, health status in canines.

## Significance

Epigenetic estimators of age (known as clocks) allow one to identify interventions that slow or reverse aging. Previous epigenetic clocks only applied to one species at a time. Here, we describe epigenetic clocks that apply to both dogs and humans. These clocks, which measure methylation levels in highly conserved stretches of the DNA, promise to increase the likelihood that interventions that reverse epigenetic age in one species will have the same effect in the other.

Ideally, model species for antiaging research should be representative of human characteristics such as size and genetic diversity, as well as shared environment. Domestic dogs (*Canis lupus familiaris*) fulfill most of these criteria, offering a unique opportunity to evaluate the effectiveness of emerging antiaging interventions (1–5). There is also a significant need to develop health-monitoring tools for dogs, as there are more than 76 million companion dogs in the United States alone (6).

Over 340 dog breeds are recognized worldwide, which are each a closed breeding population under strong selection for morphologic and behavioral traits. As a result, dogs share extensive phenotypic and genetic homogeneity within breeds and increased heterogeneity between breeds (7). Small breeds live considerably longer than large breeds (8), offering the rare chance to understand the relationship between size and lifespan within a single mammalian species. Dogs also share a similar yet accelerated trajectory of development as humans including infancy, puberty, adulthood, and senescence in about 20% of the human lifespan (5, 9). As a result, dogs represent an ideal system for studies of comparative aging, where intrabreed studies can be conducted on a background of limited diversity.

Our previous work on DNA-methylation-based age estimators (i.e., epigenetic clocks) for dogs and wolves (10) described one of the first nonhuman epigenetic clocks. We determined that the age dependence of DNA methylation (DNAm) is conserved at syntenic sites in the genomes of multiple mammalian species including humans. However, a small sample size (*n* < 150) and technical limitations associated with the measurement platform (reduced representation bisulfite sequencing) limited the generalizability of the results. Furthermore, our initial study utilized only a few canine breeds, which prevented testing the relationship between epigenetic aging and breed lifespan. Here, we report the development of a canine epigenetic clock based on 93 recognized dog breeds (11) using a mammalian array (HorvathMammalMethylChip40) that profiles highly conserved cytosines across mammalian species (12).

In this study, we present dual-species epigenetic clocks that apply to both humans and dogs. We test whether short-lived breeds exhibit faster epigenetic aging than long-lived breeds and develop epigenetic predictors of the average time to death. Finally, we investigate the relationship between breed size and lifespan and characterize 5'-C-phosphate-G-3' regions (CpGs) that are correlated with age or breed characteristics such as median lifespan or average adult weight.

## Results

### DNAm Dataset Characteristics.

We analyzed methylation profiles from 742 blood samples derived from 93 dog breeds (*Canis lupus familiaris*). Primary characteristics (sex, age, average life expectancy) for the breeds utilized are presented in Dataset S1. Median lifespans of the 93 breeds ranged from 6.3 y (Great Dane, average adult breed weight = 64 kg) to 14.6 y (Toy Poodle, average adult breed weight = 2.3 kg). Median lifespan estimates were based on the combined findings of multiple large-scale breed health publications, utilizing the median and maximum ages for each breed (*SI Appendix*, Note S1). Similar to what has been observed for roe deer (13), blood samples cluster by sex in unsupervised hierarchical cluster analysis (*SI Appendix*, Fig. S1). The clustering is due to X chromosomal CpGs that are hypermethylated in females. By contrast, an overlap between cluster structure and dog breeds could only be observed when using higher order principal components of the dataset (*SI Appendix*, Fig. S2*A*). Our attempts to create a breed classifier based on specific CpG sites using multinomial penalized regression were met with a modest 54% average correct classification rate (*SI Appendix*, Fig. S2 *B* and *C*).

### Epigenetic Clocks for Dogs and Humans.

We developed dog and human–dog epigenetic clocks using dog samples derived from blood (*n* = 742) and human samples derived from either blood or multiple tissue types (*n* = 1,352) profiled on the same mammalian array (Materials and Methods). Two distinct human–dog clocks were developed to estimate 1) chronological age in years (DNAmAge) or 2) relative age, which is the ratio of age and maximum lifespan of the respective species, between 0 and 1 (DNAmRelativeAge). This ratio allows the alignment and biologically meaningful comparison between species with very different maximum lifespans (e.g., 24 y for dog versus 122.5 y for human), which is not afforded by the simple measurement of chronological age.

**Fig. 1.**
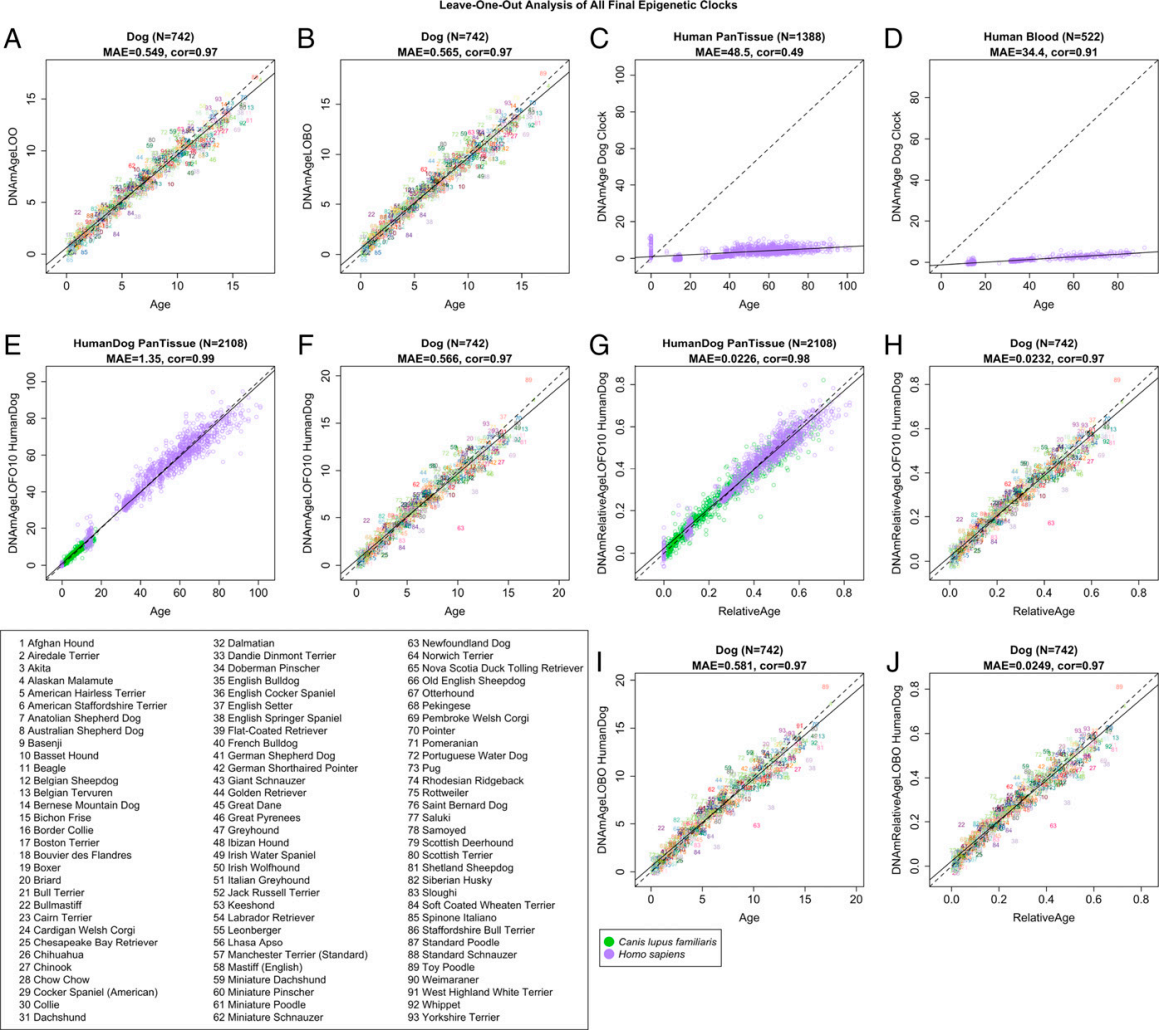
Three epigenetic clocks accurately estimate age in dogs. Evaluation of the accuracy of the pure dog clock for age (*A*–*D*), human–dog clock for chronological age (*E*, *F*, *I*), and human–dog clock for relative age (*G*, *H*, *J*). The panels differ by test set (human data, dog data, or both) and cross-validation schemes. We report three types of cross-validation schemes, as follows: LOO (*A*), LOBO (*B*, *I*, *J*), and species-balanced (LOFO10Balance) analysis of human-dog clocks for (*E* and *F*) chronological age and (*G* and *H*) relative age. Each panel reports the sample size (N), correlation coefficient (cor), and MAE.

We used cross-validation to arrive at unbiased estimates of the age correlation *R*, defined as the Pearson correlation between estimated DNAmAge and known chronological age, as well as the median absolute error (MAE; in units of years). For the pure dog clock, we observe high cross-validation estimates of the correlation (*R* = 0.97) (Fig. 1 *A*, *B*, *F*, and *H*). Different cross-validation schemes show that both the pure dog clock and the human–dog clock for chronological age exhibit a median error of less than 0.58 y (i.e., 7 mo) when using blood samples from dogs (Fig. 1 *A*, *B*, *F*, and *I*).

By definition, the pure dog clock is not expected to apply to human tissues. However, we observe a remarkably high age correlation of the dog clock using DNA from human blood samples (*R* = 0.91), albeit with a large median error of 34 y (Fig. 1*D*). The age correlation of the dog clock across all available human tissues is lower (*R* = 0.49; Fig. 1*C*) that reflects substantial confounding by tissue type.

Conversely, the human–dog clocks are designed to apply to both species (Fig. 1 *E*–*J*). The human–dog clock for chronological age has a high age correlation (*R* = 0.99) when both species are analyzed together (Fig. 1*E*) and when the analysis is restricted to dog samples alone (*R* = 0.97, Fig. 1*F*). Similarly, the human–dog clock for relative age exhibits a high correlation regardless of whether the analysis is done with samples from both species (*R* = 0.98; Fig. 1*G*) or only from dogs (*R* = 0.97; Fig. 1*H*).

To evaluate the predictive accuracy in breeds that are not part of the training data, we used a “leave one breed out” (LOBO) cross-validation strategy that divides the dataset into 93 breed groups. At each round, LOBO cross-validation trained each model on all but one breed, which was “left out” and used for validation at each iteration. LOBO cross-validation corroborated the impressive accuracy of the chronological and relative age human–dog clocks (*R* = 0.97 for both; Fig. 1 *I* and *J*).

### Epigenetic Age Acceleration Versus Breed Characteristics.

Adjusting a DNAmAge estimate for chronological age results in a measure of epigenetic age acceleration that is not correlated with chronological age (Materials and Methods). We investigated whether epigenetic age acceleration correlates with breed characteristics such as median lifespan, upper limit of lifespan, average breed weight, and average breed height, but we found no significant results (*SI Appendix*, Fig. S3). These insignificant results prompted us to develop a different type of clock that is negatively correlated with median lifespan as detailed below.

### An Epigenetic Clock Predicts Average Time to Death.

**Fig. 2.**
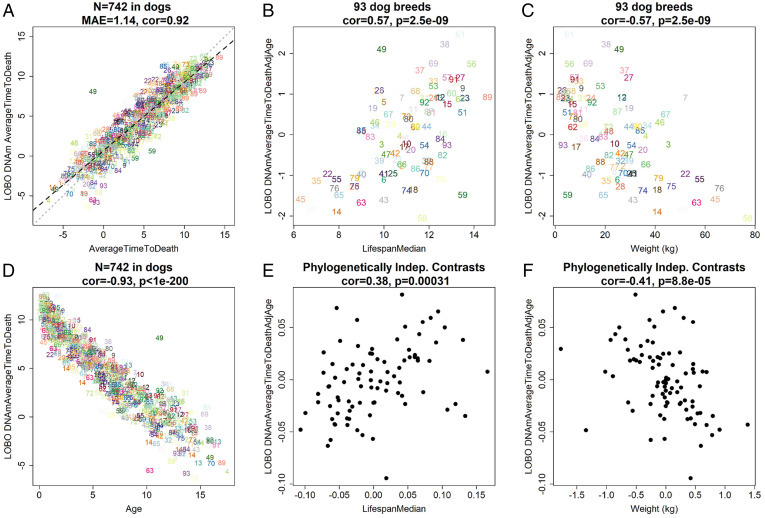
Epigenetic clocks predict average time to death. (*A*) LOBO estimates of DNAm average time to death (in years) versus average time to death in years. For each dog, the average time to death was defined as the difference between the median lifespan of the respective breed (LifespanMedian) and chronological age. (*B* and *C*) Mean of LOBO DNAm average time to death adjusted for age at breed level versus median lifespan (*B*) or weight (*C*). (*D*) LOBO DNAm average time to death versus chronological age. The association between LOBO DNAm average time to death adjusted for age and the lifespan remains significant (*P* = 1.5 × 10^−10^) even after adjusting for average adult weight in a multivariate regression model. (*E* and *F*) Phylogenetically independent (Indep.) contrast (PIC)-generated LOBO DNAm average time to death adjusted for age level versus PIC generated lifespan (*E*) or adult weight (*F*), at the breed level. For each panel, we report the sample size (*n* = 742 blood samples or *n* = 93 dog breeds), Pearson correlation estimate (cor), and Student’s *t* test *P* value. Individual dogs are colored by breed as listed in the legend of Fig. 1.

We could not develop a mortality risk predictor for dogs since we did not have follow-up information available for the individual dogs. However, we developed an epigenetic predictor of average time to death (DNAmAverageTimeToDeath) in two steps. First, we defined average time to death for individual dogs by calculating the difference between the median lifespan of the respective breed and the chronological age of the individual at the time of blood draw (AverageTimeToDeath = Lifespan Median − Age) (Materials and Methods). Second, we used elastic net regression to regress AverageTimeToDeath on DNAm across dog blood samples. LOBO analysis revealed a high correlation (*R* = 0.92; Fig. 2*A*) between AverageTimeToDeath and its DNAm-derived estimate, with an MAE of 1.14 y. We expect that this model can be extrapolated to breeds not included in our dataset as the LOBO analysis was successful across 93 distinct breeds.

We observe that age-adjusted DNAmAverageTimeToDeath correlates in the expected direction with median lifespan (*R* = 0.57 and *P* = 2.5 × 10^−9^; Fig. 2*B*) and average adult breed weight (R = −0.57 and *P* = 2.5 × 10^−9^; Fig. 2*C*). Similarly, relationships can be observed even after adjusting for phylogeny (Fig. 2 *E* and *F*). As expected from its construction, DNAmAverageTimeToDeath has a strong negative correlation with chronological age (R = −0.93; Fig. 2*D*).

This is consistent with the fact that younger dogs are further from the median lifespan of their respective breed than older dogs. A multivariate regression model shows that the association of DNAmAverageTimeToDeath with median lifespan is retained even after adjusting for chronological age, sex, and average adult weight of the breed (*P* = 1.5 × 10^−10^; Table 1). The association remained significant after adjusting for phylogenic relationships using the phylogenetic independent contrast method (*P* = 1.1 × 10^−4^; Table 1) (Materials and Methods).

**Table 1.** Linear regression analysis for dog breed lifespan

	**β**	**SE**	***P***
Model 1: Dependent variable–median lifespan			
Intercept	12.377	0.114	<2.0E-16
Age-adjusted DNAmAverageTimeToDeath	0.248	0.038	1.5E-10
Weight (kg)	−0.060	0.004	3.2E-48
Female	−0.029	0.101	0.8
Model 2: Dependent variable–PIC median lifespan			
Intercept	11.795	0.309	<2.0E-16
PIC Age-adjusted DNAmAverageTimeToDeath	0.719	0.178	1.1E-4
PIC Weight (kg)	−0.035	0.011	2.0E-3

Model 1 presents the results from linear regression model analysis of dog breed median lifespan (dependent variable) on age-adjusted DNAm average time to death (in years), sex, and breed weight (kg) at individual dog level. Model 2 presents the results from the PIC regression analysis that accounts for phylogenetic relationships between breeds. PIC median breed lifespan (dependent variable) was regressed on PIC age-adjusted DNAm average time-to-death and PIC breed weight. Columns indicate the covariate name, regression coefficient, SE, and two-sided Wald test *P* value.

As an additional validation step, we split the blood samples into two sets, namely, the training set consisted of *n* = 571 dogs (77%) and a test set consisted of *n* = 171 (23%) dogs, which were balanced by breed (Materials and Methods). The training data were used to define DNAmAverageTimeToDeath as outlined above. In the test data, we observed a high correlation (*R* = 0.95) and a relatively low MAE (0.82 y) between AverageTimeToDeath and its DNAm-based estimate (*SI Appendix*, Fig. S4*A*). To address the concern that our analysis was biased by a disproportionate number of Portuguese water dogs (PWDs; *n* = 95 in our entire dataset), we randomly downsampled the breed to only 5 dogs, resulting in a smaller training set (*n* = 505 including 5 PWDs). Again, we found comparable results (*SI Appendix*, Fig. S5).

### Epigenome-Wide Association Study (EWAS) of Age in Dogs and Humans.

**Fig. 3.**
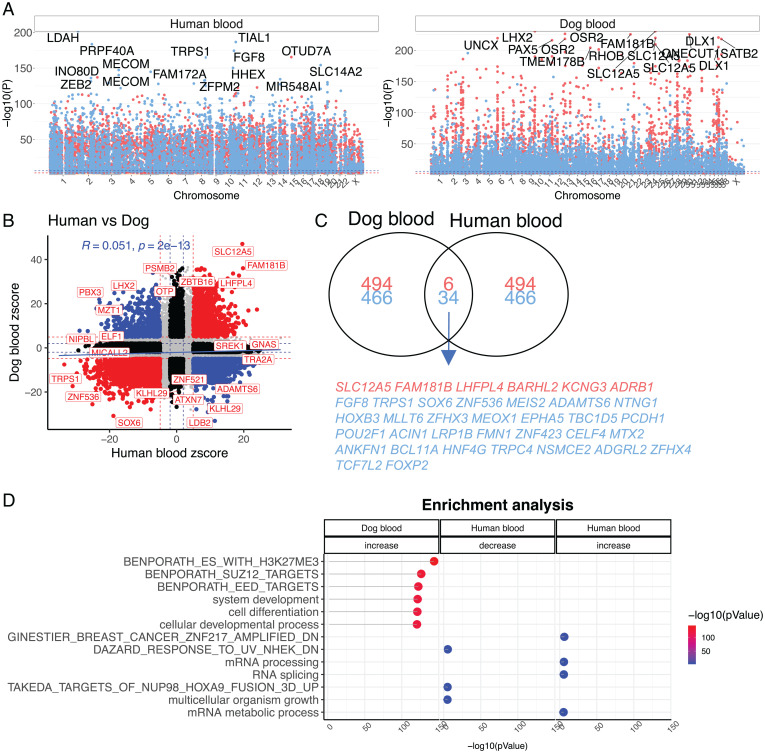
EWAS of chronological age in humans and dogs. (*A*) Manhattan plots of EWAS of age in humans (*n* = 508, 12.5 to 92.04 chronological y) and dogs (*n* = 742, 0.1 to 17.5 chronological y). The analysis was limited to 20,622 CpGs that aligned to orthologous genes in both species. The EWAS was carried out using a correlation test (Fisher transformation of Pearson correlation) between cytosine methylation and age. The red and blue dashed lines correspond to significance levels of *P* = 0.001 and *P* = 10^−6^, respectively. (*B*) Scatter plot of aging effects (correlation test Z statistics) in humans versus dogs. The Z statistics was calculated by applying the Fisher z-transformation to the Pearson correlation between CpG methylation and age for each species. Positive and negative values of the Z statistic indicate an age-related increase or decrease in methylation, respectively. Red dots indicate shared CpGs, black dots indicate species-specific CpGs, and blue dots indicate divergent changes. (*C*) Venn diagram of the overlap of top 1,000 CpGs (500 per direction) in both species. (*D*) GREAT enrichment analysis (15) of top 500 age-related CpGs per direction in each species. Column headings specify age-related decrease or increase in methylation. GREAT analysis was limited to 20,622 probes conserved between humans (Hg19) and dogs (CanFam_Great Dane v.3.1). We provide justification for the use of the GREAT analysis framework in the *SI Appendix*, Notes S4 and S5. The reported terms are significant at *P* < 1 × 10^−5^. Analyzed datasets were gene ontology biological processes and MsigDB perturbation.

In total, 31,911 CpG probes on the mammalian methylation array (5,021 genes) map to the genome assembly of the Great Dane (CanFam_GreatDane.UMICH_Zoey_3.1.100) (14). The mammalian array has high interspecies conservation and is expected to apply to all dog breeds (12). For the human–dog analysis, we limited the probes to 20,622 CpGs that map to orthologous genes between both species. As expected, age had a strong effect on DNAm levels in dogs; 9,625 (46% of total) CpGs significantly correlated with age (Bonferroni-corrected *P* < 1 × 10^−6^) (Fig. 3*A* and Dataset S2). Top age-related dog CpGs are located in an intron of *SLC12A5* (correlation test Z statistic, z = 46), an intron of *LHX2* (z = 36), and an exon of *OSR2* (z = 36). In general, CpGs that gained methylation with age were located near polycomb repressive complex 2 (PRC2) targets (e.g., genes with the trimethylated H3K27 mark in their promoters in human embryonic stem cells) and enriched with genes that play a role in development (Fig. 3*D* and Dataset S3).

Age effects on individual cytosine methylation in dog blood correlate only weakly with those of human blood (*R* = 0.051; Fig. 3*B*). The low correlation may reflect true species differences or differences in the distribution of chronological age with respect to very young ages; human samples ranged from 0.1 to 0.75 relative age (12.5 to 92 actual human years, relative to a maximum human lifespan of 122.5 y), whereas dog samples ranged from 0.0044 to 0.73 relative age (0.1 to 17.5 actual dog years, relative to a maximum dog lifespan of 24 y). Comparing the age effects on dog blood to aggregated human tissues resulted in a stronger (albeit still mild) correlation (*R* = 0.21; *SI Appendix*, Fig. S6*A*). Despite the low correlation between EWAS results, a small subset (40 CpGs) of the most significant 1,000 CpGs displays a similar aging pattern between dogs and humans (Fig. 3*C*). Interestingly, the top CpG-associated gene in dogs (*SLC12A5*) is also related to age in humans (z = 19.5). Shared CpGs positively associated with aging are found in the following genes: *SLC12A5*, *FAM181B*, *LHFPL4*, *BARHL2*, *KCNG3*, and *ADRB1*. Shared CpGs negatively associated with aging are found in genes including *FGF8*, *TRPS1*, *SOX6*, *ZNF536*, *MEIS2*, and *ADAMTS6*. Genomic region enrichment annotation (GREAT) analysis of EWAS of age in humans implicated that genes whose methylation associates with aging in either direction relate to mRNA processing (Fig. 3*D*).

### EWAS of Breed Characteristics.

Dog breeds have a unique and intriguing inverse relationship between average adult weight and life expectancy, with smaller dogs living up to twice as long as larger breeds (16). This contradicts observations about size and lifespan at the species level and may be due in part to domestication and artificial selection (8). We hypothesized that an EWAS of lifespan, weight, and height could reveal the identities of determinant genes and biological processes that couple growth with lifespan in domestic dogs.

**Fig. 4.**
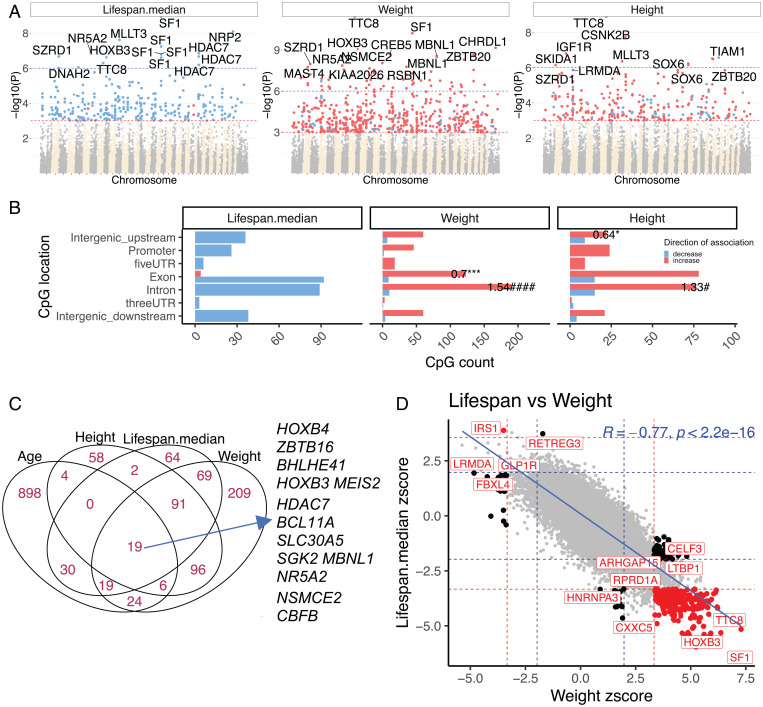
EWAS of breed characteristics. (*A*) Manhattan plots of the EWAS results. Coordinates were estimated based on the alignment of the mammalian array probes to the CanFam_GreatDane.UMICH_Zoey_3.1.100 genome assembly. Red dots correspond to a positive association between DNAm at a given CpG and each breed trait, whereas blue dots represent a negative correlation. The red dashed line indicates a significance threshold of *P* < 10^−3^; the blue line corresponds to the Bonferroni-corrected significance level of *P* < 1 × 10^−6^. The top 15 CpGs were labeled according to their neighboring genes. (*B*) Location of the top CpGs in each tissue relative to the closest gene transcriptional start site. Enrichment per location type was compared to the assay background using Fisher’s exact test. The numbers on bars indicate proportion change OR. *P* < 0.05 (*negative OR, ^#^positive OR), ***P < 0.001, ^####^*P* < 0.0001. (*C*) The Venn diagram shows the overlap of top CpGs associated with chronological age, breed median lifespan, average weight, and average height. Student’s *t* test statistic for age was used to select the top 500 positively and top 500 negatively age-related CpGs. (*D*) Sector plot of EWAS of breed lifespan versus weight. Red dots mark shared significant CpGs; black dots indicate significant trait-specific CpGs. Dashed lines indicate the significance threshold: *P* < 1 × 10^−3^ (red) and *P* > 0.05 (blue). The Z scores are Fisher’s z-transformation of Pearson correlation coefficients for each outcome.

Considering the unbalanced number of dogs per breed in the dataset, we averaged the methylation levels of each CpG by breed. We carried out two statistical modeling approaches, as follows: 1) a primary analysis relating CpGs to breed characteristics without accounting for phylogenetic relationships between breeds and 2) a secondary analysis that adjusts for phylogenetic relations between breeds and/or breed weight. In our primary EWAS analysis, we found 16 CpGs that associate with median lifespan (Bonferroni-corrected *P* < 1 × 10^−6^), 67 CpGs that relate to adult breed weight, and 10 CpGs that relate to adult breed height (Fig. 4*A*). Details on these CpGs are presented in Dataset S4. Top CpGs for each EWAS include median lifespan, *SF1* exon (z = −6.3); breed weight, *TTC8* intron (z = 7.2); and breed height, *TTC8* intron (z = 6.2).

Since larger sets of input CpGs are needed to properly power enrichment analyses, we lowered the significance threshold to *P* < 1 × 10^−3^. Most CpGs that associate with lifespan (*P* < 1 × 10^−3^) exhibit decreased methylation regardless of their genomic location (Fig. 4*B*). In contrast, CpGs associated with either breed weight or height exhibit increased methylation (Fig. 4*B*). Nineteen significant CpGs are shared among breed lifespan, breed weight, breed height, and chronological age (Fig. 4*C*).

EWAS results for median lifespan negatively correlate with those for breed weight (R = −0.77, Fig. 4*D*), reflecting the inverse relationship between breed weight and median lifespan. Hence, a CpG that correlates positively with lifespan is expected to exhibit a negative correlation with weight (and vice versa) (*SI Appendix*, Fig. S7). As expected, adjusting for phylogeny or breed weight led to fewer significant findings (*SI Appendix*, Fig. S8).

### Dog EWAS Versus Human GWAS.

**Fig. 5.**
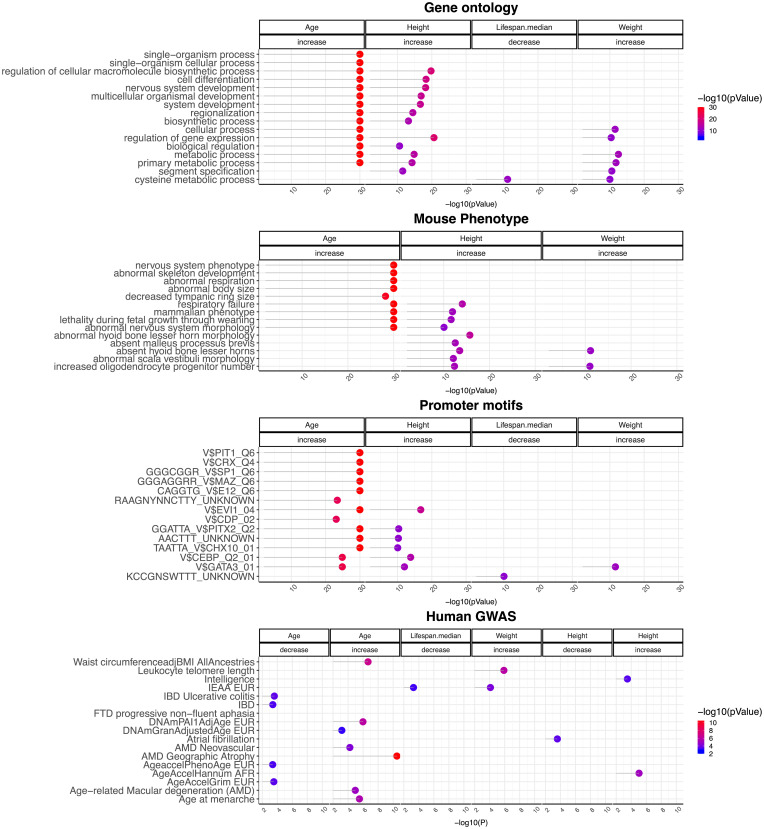
Gene set enrichment analysis of CpGs related to age, breed lifespan, and breed weight of dogs. Gene set enrichment analysis was conducted on 20,622 CpGs that map to orthologous genes of the human Hg19 and Great Dane genome assembly using the GREAT tool (15). Each panel represents the top two enriched datasets from each category (gene ontology, mouse phenotypes, promoter motifs, and MsigDB perturbation) (*P* < 10^−10^). *P* values were log-transformed for easier visualization. The panel for human GWAS enrichment represents the significant results from the genomic-region-based enrichment analysis between 1) the top 2.5% genomic regions involved in GWAS of complex traits-associated genes and 2) up to the top 500 increased/decreased CpGs for each EWAS.

To uncover correlations between evolutionarily conserved cytosine variants in dogs and known human traits, the proximal genomic regions of the top positive and negative CpGs (up to 500 in each direction) identified via EWAS were intersected with the top 2.5% of genes associated with several human traits according to genome-wide association studies (GWASs; *Materials and Methods* and *SI Appendix*, Fig. S3). A hypergeometric test analysis was performed at the level of genomic regions (as opposed to CpGs) to avoid confounding due to gene size or number of CpGs within a gene in our methylation array. The proximal genes of age-related CpGs significantly overlapped with human genes identified in GWAS of human epigenetic age acceleration according to the blood-based clock by Hannum et al. (17) and the DNAm-based mortality risk predictor GrimAge clock (18), age-related macular degeneration (19), and age at menarche (20, 21) (Fig. 5 and Dataset S5). EWAS of dog breed lifespan, breed weight, and breed height did not overlap strongly with human GWAS results, except for EWAS of breed weight and human GWAS of leukocyte telomere length (*P* = 1.2 × 10^−6^).

Further similarity between the epigenetics of dogs and humans is evident by the overlap of age-related canine genes and those implicated in human maternal longevity, age at menarche, educational attainment, frontotemporal dementia, age-related macular degeneration, and mortality risk as measured by GrimAge clock (12). We observed a nominally significant overlap (*P* = 0.01) between genes implicated by the EWAS of dog breed lifespan with those implicated by GWAS of human parental lifespan, based on the following shared genes: *TUBG1*, *FAM134C*, *BCL11A*, *PRPF40B*, *HIPK1*, *DYNLRB2*, *FIGN*, *PGS1*, and *TOX* (Dataset S5).

### DNAm Patterns in Genes Identified by GWAS of Dog Body Weight and Lifespan.

Previous GWAS studies highlighted a small number of genes including *IGSF1*, *IGF1R*, *ASCL4*, *IGF1*, *LCORL*, *HMGA2*, *GHR*, *CD36*, *SMAD2*, *IGF2BP2*, and *ESR1* with variants that explain 64 to 95% of body weight variability in breeds tested (3, 22–24). Doherty et al. (24) describe a genetic variant within *PRDX1* that was marginally significantly associated with longevity residuals that had been corrected for the effects of body weight. Of these genes of interest, the mammalian methylation array contains 47 probes adjacent to different regions of *IGF1*, *ESR1*, *SMAD2*, *HMGA2*, *IGF1*, *IGF2BP2*, *IGSF1*, and *IGF1R* (Dataset S2). Only four CpGs located downstream of *SMAD2* and one CpG in exon 2 of *IGF1R* positively correlate with adult weight and negatively correlate with breed lifespan (Dataset S2), suggesting a modest epigenetic regulation of the inverse relationship between size and lifespan in adult dogs, at least in these GWAS-implicated genes (25).

### Chromatin State Analysis and PRC Targets.

**Fig. 6.**
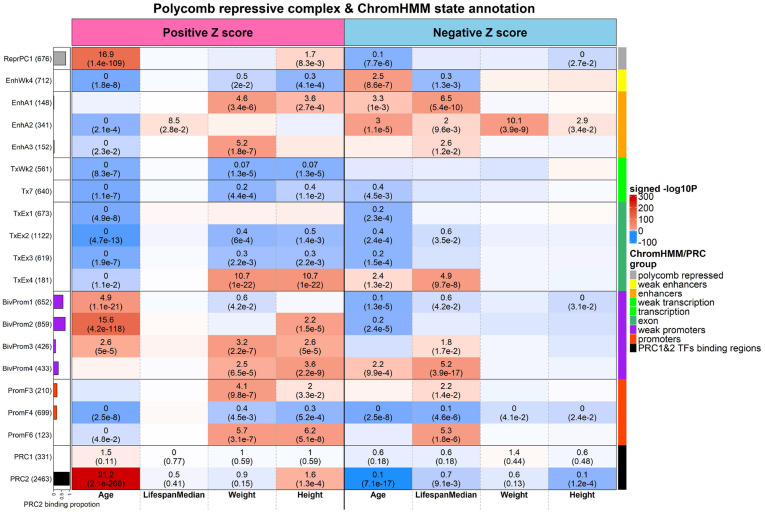
Hypergeometric overlap analysis between the top CpGs identified in our EWAS studies (columns) and two panels of CpGs (rows), as follows: 1) universal chromatin state analysis (26) and 2) PRC1 and PRC2 binding from ENCODE ChipSeq datasets (27). We display 18 universal chromatin states that show significant enrichment/depletion in EWAS of age, median breed lifespan, breed weight, or breed height (hypergeometric *P* < 1.0 × 10^−6^) and the PRC annotations. For each EWAS, we annotated up to the top 500 CpGs with positive and negative Z scores, respectively (*P* < 0.01). Each cell indicates the OR (*Top* value) and *P* value (*Bottom* value). The cell color is based on −log10 (*P* value) multiplied by the sign of OR > 1. Red denotes OR > 1; blue denotes OR < 1. The barplot (at *y* axis) depicts the proportion of PRC2 binding ranges from zero to one. The legend indicates chromatin states based on their group category and PRC group. The *y* axis lists chromatin state or PRC binding category with the number of mammalian array CpGs inside parentheses.

Finally, we interpret our EWAS results in light of a universal chromatin state map, which defined 16 groups of chromatin states from 1,032 experiments mapping chromatin marks across 127 human cell and tissue types, including active and weak enhancers (EnhA, EnhW), bivalent states associated with promoters (BivProm), flanking promoter states (PromF), polycomb repressed states associated with H3K27me3 (ReprPC), and states associated with exons and transcription (TxEx). (Dataset S6) (26). We analyzed up to 500 CpGs with the top positive and negative Z scores (*P* < 0.01) from our EWAS results with chromatin state as well as polycomb repressive complex 1 (PRC1) and PRC2 (*Materials and Methods*). Enrichment was first noted for the overlap between positively age-related CpGs and PRC2 binding sites (odds ratio [OR] = 31.2, *P* = 2.1 × 10^−266^, Fig. 6 and Dataset S7).

PRC2 is a transcriptional repressor complex best known as a writer of H3K27 methylation, a chromatin mark associated with transcriptional repression (28). Previous studies showed its association with gain of methylation across different tissue types in human tissues (29, 30) and in more than 110 mammalian species (31). Interestingly, chromatin states that significantly overlapped with positively age-related CpGs contained a high proportion of CpGs in PRC2 binding sites, such as BivProm2 (OR = 15.6, *P* = 4.2 × 10^−118^), BivProm1 (OR = 4.9, *P* = 1.1 × 10^−21^), and ReprPC1 (OR = 16.9, *P* = 1.4 × 10^−109^). The bivalent chromatin state BivProm2 is associated with the active promoter mark H3K4me3 and the repressive mark H3K27me3, in addition to being located in a PRC2 binding region (26). In contrast, the CpGs exhibiting a negative correlation with breed lifespans are enriched with BivProm4 (OR = 5.2, *P* = 3.9 × 10^−17^), TxEx4 (OR = 4.9, *P* = 9.7 × 10^−08^), PromF6 (OR = 5.3, *P* = 1.8 × 10^−6^), and EnhA1 (OR = 6.5, *P* = 5.4 × 10^−10^). Echoing the negative correlation between breed lifespan and body size (25), the chromatin state overlap patterns of CpGs associated with breed weight and height inversely mirror the overlap patterns of breed lifespan (Fig. 6). For instance, PromF6 enrichment is associated with the CpGs positively correlated with dog weight (OR = 5.7, *P* = 3.1 × 10^−7^) and height (OR = 6.2, *P* = 5.1 × 10^−8^) and CpGs negatively correlated with lifespan (OR = 5.3, *P* = 1.8 × 10^−6^) and further lacks CpGs targeted by PRC2 binding.

## Discussion

Epigenetic aging clocks were first developed for humans (17, 32, 33). In a short time, their reported use covered a large swath of medical and scientific research areas (34, 35). It quickly became clear that these clocks capture many important features of the biological aging process. They readily found their way into biomedical applications including human clinical trials (36). More recent human epigenetic clocks are able to predict mortality risk in a manner believed to be independent of all other established aging biomarkers (34). Several mouse epigenetic clocks have since been developed and successfully validated against putative longevity treatments or genetic interventions such as rapamycin, caloric restriction, and growth hormone receptor knockout models (37–42). Each of these treatments demonstrates a significant regression of epigenetic aging in mice, supporting the notion that epigenetic clocks can be used to rapidly test the effectiveness of antiaging interventions.

There is no guarantee that an intervention that reverses epigenetic age in an animal model will successfully translate to humans. However, the likelihood of success increases substantially if a suitable animal model like the dog is used to measure a conserved biomarker of aging, such as a human–dog clock. To create such a third generation epigenetic clock that crosses the species barrier, we used the mammalian DNAm array that profiles CpGs embedded within sequences that are conserved across mammals (12). This array platform has been used to develop epigenetic clocks for cats, sheep, elephants, primates, and many other mammalian species (43–46). Our prior work on dogs, wolves, and humans demonstrated that age associations of syntenic CpGs were conserved between canids and humans, even though the data were generated on different platforms (10). Here, we developed dual-species human–dog clocks that relate relative age to cytosine methylation. These dual-species clocks accurately estimate chronological age of dogs and humans using the same mathematical formula, highlighting yet again the common underlying mechanisms of aging between mammals.

Recently, Wang et al. (5) developed an oligo-capture system to characterize the canine DNA methylome, targeting syntenic regions of the genome. The authors present an epigenetic clock that was trained in one species, dog, but leads to a moderately high correlation in another species (i.e., mouse–dog clock, age correlation *R* = 0.73). In contrast, our human–dog clocks were trained in both species (humans and dogs) simultaneously, which may explain the substantially higher age correlation (*R* = 0.97). Other factors such as validation schemes, sample sizes, sample preparation methods, and technical variability may impact results as well.

The chronological age clocks are not associated with breed characteristics such as median lifespan or breed weight. To address this limitation, we also developed an epigenetic estimator of average time to death, which potentially estimates mortality risk for individual dogs based on blood methylation profiles. However, this predictor requires validation.

While our clocks were trained in blood, we expect that they produce high age correlations with saliva or buccal samples as well. However, a constant offset term (difference between DNAmAge and age) may need to be derived for these sample types and other alternative sources of DNA to enable a more routine and convenient sample collection.

Breed identity is not accurately recapitulated by DNAm and has a much weaker effect on methylation compared with sex and age. However, we note that breeds with more samples tend to cluster together more tightly (*SI Appendix*, Fig. S2*A*), suggesting that increasing the sample size could lead to a better breed classification. Moreover, unaccounted confounders such as cell fraction proportions could preclude breed identification.

The inverse relationship between size and lifespan, an intriguing feature of dogs, could prove enlightening for humans if it were understood at the molecular level (8). Disappointingly, our epigenetic clocks for chronological age (Fig. 1) did not relate to breed weight or breed lifespan. This motivated us to build an another epigenetic biomarker (DNAmAverageTimeToDeath) that predicts average time to death and exhibits positive and negative correlations with breed lifespan and weight, respectively (Fig. 2). CpGs that correlate with breed average weight were found in close proximity to genes involved in human adipogenesis, and age-related CpGs overlapped with waist-to-hip ratio genes in humans (Dataset S5). The finding that canine CpGs that gain methylation with age are located in CpG islands near targets of PRC2 and developmental genes is also consistent with findings in humans (29, 30, 47). This highlights the increasingly frequent observation that the process of development is connected to epigenetic aging. There is no further empirical data to develop a focused hypothesis, but the availability of epigenetic clocks promises to remedy this.

Of previously highlighted canine weight and lifespan-related genes (3, 22–24), *SMAD2*, and *IGF1R*-associated methylation positively related to average breed weight but inversely related to breed lifespan. Several studies have shown that aging effects on methylation are conserved at specific locations that play a role in mammalian development (reviewed in 34, 48). The substantial number of methylated loci with opposing correlations between breed weight and breed lifespan in these and novel genes provide an opportunity to investigate this phenomenon. Beyond our application to aging, the methylation data may lend themselves for studying the epigenetic effects of domestication (49).

Collectively, the successful development of epigenetic clocks for dogs and the dual-species clocks, in particular, underlie the universality of the epigenetic aging process. It demonstrates that, at least at the DNA level, there are remarkable similarities in the aging process between dogs and humans. Finally, our EWAS results highlight gene regions that may underlie the inverse relationship between breed weight and lifespan.

## Materials and Methods

### Materials.

DNA samples from *n* = 742 dog blood samples from 93 breeds (Dataset S1) were provided by researchers at the National Human Genome Research Institute (NHGRI). The weight of individual dogs was unknown. The collection was approved by the Animal Care and Use Committee of the Intramural Program of NHGRI at the National Institutes of Health (protocol #8329254).

### Lifespan and Breed Characteristics.

Standard breed weight (SBW), standard breed height (SBH), and lifespan were aggregated from several sources. SBW and SBH were taken from previously reported values (23, 50), which were updated if American Kennel Club (AKC) values differed (16). If the AKC did not specify SBW or SBH, we used data from Atlas of Dog Breeds of the World (51). Lifespan estimates were calculated as the average of the standard breed across sexes, compiled from numerous publications consisting primarily of multibreed surveys of age and cause of death from veterinary clinics and large-scale breed-specific surveys, which are often conducted by purebred dog associations. Sources for lifespan are reported in *SI Appendix*, Note S1. When available, data were combined across surveys for the number of dogs and minimum, maximum, mean, and median age at death. The minimums, maximums, and medians were averaged across studies to produce a representative lifespan expectation for each breed. For three breeds (American hairless terrier, sloughi, and Ibizan hound), no published survey data were available. For these breeds, the maximum age expectation was obtained from the AKC website.

### Human Tissue Samples.

To build the human–dog clock, we analyzed previously generated methylation data from *n* = 1,352 human tissue samples (adipose, blood, bone marrow, dermis, epidermis, heart, keratinocytes, fibroblasts, kidney, liver, lung, lymph node, muscle, pituitary, skin, spleen) from individuals aged 0 to 93 y old. Samples came from three sources, namely, tissue and organ samples came from the National NeuroAIDS Tissue Consortium (NNTC) (52, 53); blood samples from the Cape Town Adolescent Antiretroviral Cohort study (54); and blood, skin and other primary cells were provided by Kenneth Raj (55). All were obtained with Institutional Review Board (IRB) approval (IRB#18-000315).

### DNAm Data.

Both dog and human data were generated on the same platform (HorvathMammalMethylChip40) (12). The mammalian array provides high coverage of up to 36K highly conserved CpGs in mammals (12). A total of 31,911 CpGs that map to the Great Dane assembly (CanFam_GreatDane.UMICH_Zoey_3.1.100) were used in the analysis. Genome coordinates for different dog breeds are posted on the Mammalian Methylation Consortium Github (*Data Availability*). The chip manifest file can be found at Gene Expression Omnibus at National Center for Biotechnology Information (NCBI) as platform GPL28271. The SeSaMe normalization method was used to define beta values for each probe (56).

### Penalized Regression Models.

Technical details and R software code are presented in *SI Appendix*, Note S3. We developed four different epigenetic clocks for dogs by regressing the outcome (chronological age, relative age, or average time to death) on all CpGs that map to the Great Dane assembly. All tissues were used for the pantissue clock, whereas the analysis for the tissue-specific clocks (blood, liver, and brain) was restricted to the respective tissue only. Penalized regression models were created with the R function “glmnet” (57). By definition, the α-value for the elastic net regression was set to 0.5 (midpoint between Ridge and Lasso type regression) and was not optimized for model performance. The optimal penalty parameters in all cases were determined automatically by using 10-fold internal cross-validation (cv.glmnet) on the training set. We performed a cross-validation scheme for arriving at unbiased or least biased estimates of the accuracy of the epigenetic clocks, consisting of leaving out a single sample from the regression (leave-one-out [LOO]), predicting an age for that sample, and iterating over all samples. The covariates (CpGs) and coefficient values of the different multivariate regression models can be found in Dataset S8. The latter also specifies the genomic locations of the CpGs in two genomes, as follows: GreatDane.UMICH_Zoey_3.1.100 and Canis familiaris 3.1.

### Relative Age Estimation.

To enable a comparison of species with very different lifespans, as well as to overcome the inevitable skewing due to unequal distribution of data points from dogs and humans across age range, relative age estimation was made using the formula relative age = age/max lifespan, where the maximum lifespan for dogs and humans were set to 24 y and 122.5 y, respectively (AnAge) (58). The oldest dog ever verified lived 29 y and 5 mo according to the Guinness Book of World Records. For our purposes, there is no difference between a maximum lifespan of 24 or 29 or lower. For the sake of consistency with other studies from the Mammalian Methylation Consortium, we are using the value (24 y) reported in the database anAge.

### Measures of Epigenetic Age Acceleration.

The predicted value resulting from an elastic net regression model of age is denoted as “DNAmAge.” By definition, DNAmAge is highly correlated with chronological age. To remove the confounding effect of age, we regressed DNAmAge on chronological age and formed raw residuals. The resulting residuals are not correlated with chronological age (*R* = 0). A positive or negative value of epigenetic age acceleration indicates that the DNAmAge estimate is higher or lower than expected versus chronological age. Similarly, we carried out age adjustments for DNAm based on estimates of relative age or average time to death.

Cross-validation was carried out with LOO, LOBO, and species-balanced (LOFO10Balance) methods. LOO and LOBO cross-validation trained each model on all but one individual or breed, respectively. The left out individual/breed was then used as a test set. LOFO10Balance was implemented by partitioning both the combined human/dog dataset into 10 evenly sized folds, where each fold has the same proportion and human and dog samples (referred to as balanced folds). We then iterated through each fold, training on the other nine folds, and applied the model to the target fold.

### EWASs of Age, Lifespans, and Weight.

EWAS was performed in each tissue separately using the function standardScreeningNumericTrait from the WGCNA R package (59).

### Epigenetic Clock for Average Time to Death.

Follow-up time-to-death data were not readily available for individual dogs in our study. To create a surrogate variable for this important endpoint, we leveraged two known variables, as follows: 1) the median lifespan per breed and 2) the chronological age at the time of the blood draw. For each dog, we defined AverageTimeToDeath as the difference between median breed lifespan and chronological age.

To protect against confounding by age, we used two approaches. First, we defined age-adjusted measures as raw residuals resulting from regressing the DNAm-based biomarker on chronological age. For example, we regressed DNAmAverageTimeToDeath (dependent variable) on age using ordinary least squares regression. Next, we defined the age-adjusted measure as residual (actual value minus predicted value). The resulting residual turns out to be uncorrelated with chronological age. Second, we used multivariate regression models where chronological age was added as the covariate.

To assess the accuracy of elastic net regression models (R function glmnet), we used LOBO cross-validation, training each model on all but one breed. The left out breed was then used as a test set. The LOBO approach assesses how well the penalized regression models generalize to breeds that were not part of the training data. To ensure unbiased estimates of accuracy, all aspects of the model fitting including prefiltering CpGs were conducted in the training data for the LOBO analysis. We fit the glmnet model to the top 6,000 CpGs with the most significant median Z score (lifespan correlation test) in the training data. The top 6,000 CpGs were selected by averaging the CpG values for each breed and conducting EWAS of the median lifespan.

### Phylogenetically Independence Contrast Analysis.

We computed phylogenetically independence contrasts (PICs) (60) for lifespan and adult weight DNAm biomarkers using the R package ape (61). Individual-level data such as age-adjusted DNAmAverageTimeToDeath were averaged by dog breed to conduct PIC analysis on breed level data.

### Sensitivity Analysis for DNAmAverageTimeToDeath.

We randomly split the sample into training (75%) and test dataset (25%) at the breed level. As a result, the training (*n* = 571) and test (*n* = 171) dataset are balanced in the breed level. Both the training and test dataset contain 93 dog breeds. We performed an elastic net analysis (α = 0.5) to build DNAmAverageTimeToDeath using the training dataset and evaluated its prediction accuracy on the test dataset including its correlation with AverageTimeToDeath, chronological age, and dog breed character. We also repeated the PIC analysis. To address concerns that our analysis was confounded by a disproportionately large number of PWDs (*n* = 71 in the training and *n* = 24 in the test dataset), we randomly removed 66 PWD samples from the training dataset such that only 5 PWD samples remained in the training dataset. As a result, each breed was represented by about five animals each. The analysis was repeated on the revised training (*n* = 505, 5 PWDs) and test dataset (*n* = 171).

### EWAS-GWAS–Based Overlap Analysis.

EWAS-GWAS overlap analysis related gene sets found by our EWAS of age with gene sets identified by published large-scale GWAS of various phenotypes, including body fat distribution, lipid panel outcomes, metabolic outcomes, neurological diseases, six DNAm based biomarkers, and other age-related traits (Dataset S5). Data from 102 GWAS were utilized (*SI Appendix*, Note S3). The six DNAm biomarkers included four epigenetic age acceleration measures derived from 1) Horvath’s pantissue epigenetic age adjusted for age-related blood cell counts referred to as intrinsic epigenetic age acceleration (33, 62); 2) Hannum’s blood-based DNAm age (17); 3) DNAmPhenoAge (63); and 4) the mortality risk estimator DNAmGrimAge (18), as well as with DNAm-based estimates of 5) blood cell counts and 6) plasminogen activator inhibitor 1 levels (18). For each GWAS result, we used MAGENTA software (64) to calculate an overall GWAS *P* value per gene, which is based on the most significant single nucleotide polymorphism (SNP) association *P* value within the gene boundary (±50 kb) adjusted for gene size, number of SNPs per kb, linkage disequilibrium pattern, and other potential confounders (64). For each EWAS result, we studied the genomic regions from the top 1,000 CpGs (500 per direction of association) with dog relevant traits, as follows: lifespan, weight, lifespan adjusted weight, and chronological age. For those, the top 633 CpGs had an EWAS of *P* < 0.05. To assess the overlap with a test human trait, we selected the top 2.5% genes for each GWAS trait and calculated one-sided hypergeometric *P* values based on genomic regions as detailed in *SI Appendix*, Note S3. The number of background genomic regions in the hypergeometric test was based on the overlap between the entire genes in a GWAS and the entire dog genomic regions (∼32k CpGs) in our mammalian array. We caution the reader that the analysis was not adjusted for multiple comparisons.

### PRC Region Overlap Analyses.

PRC annotations were defined based on the binding of at least 2 transcriptional factor members of PRC1 (RING1, RNF2, BMI1) or PRC2 (EED, SUZ12, and EZH2) in 49 available chromatin immunoprecipitation sequencing (ChipSeq) datasets in ENCODE (27). Using the 20,622 probes that aligned to orthologous genes in both human and dog species, we identified 331 and 2,463 CpGs on the array that were located in regions bound by PRC1 and PRC2, respectively. We performed one-sided hypergeometric analysis to study the overlap between PRC1 or PRC2 regions, using up to the top 500 CpGs with positive and negative Z scores (*P* < 0.01) from the EWAS of age, median lifespan and weight and height of dog breed. We report both the enrichment (OR > 1) and depletion (OR < 1) patterns for the overlap analysis.

### Universal Chromatin State Analysis.

To annotate the EWAS-generated CpGs sets based on chromatin states, we assigned a state for the 20,622 CpGs based on the universal ChromHMM chromatin state annotation of the human genome (26). The underlying hidden Markov model (HMM) was trained using chromatin mark data gathered from 1,053 datasets and 127 cell and tissue types to produce a single annotation of the genome per position. A total of 100 distinct states were generated and categorized into 16 major groups according to the parameters in the HMM model and genome annotations (Dataset S6). Of the total 100 states, our 20,622 CpGs map to 97 distinct states. Similar to the overlap analysis with PRC regions, we performed one-sided hypergeometric analysis to study both the enrichment (OR > 1) and depletion (OR < 1) patterns of EWAS CpGs across the chromatin states.

Additionally, we note the following URLs: American Kennel Club, http://www.akc.org/dog-breeds/; AnAge, http://genomics.senescence.info/help.html#anage; UCSC genome browser, http://genome.ucsc.edu/index.html; CanFam_GreatDane.UMICH_Zoey_3.1.100 Assembly, https://www.ncbi.nlm.nih.gov/assembly/GCF_005444595.1/.

## Supplementary Material

Supplementary File

Supplementary File

Supplementary File

Supplementary File

Supplementary File

Supplementary File

Supplementary File

Supplementary File

Supplementary File

## Data Availability

The data are publicly available as part of the data release from the Mammalian Methylation Consortium. Genome annotations of these CpGs can be found on GitHub (https://github.com/shorvath/MammalianMethylationConsortium) (65). The mammalian methylation array (HorvathMammalMethylChip40) can be purchased from the nonprofit Epigenetic Clock Development Foundation (https://clockfoundation.org/). All other study data are included in the article and/or supporting information.
