# Supplementary material for: DNA methylation clocks for dogs and humans
Source: Proc Natl Acad Sci U S A. 2022 May 17;119(21):e2120887119. doi: 10.1073/pnas.2120887119 (PMC9173771; doi:10.1073/pnas.2120887119)
Supplement: Supplementary File [file pnas.2120887119.sapp.pdf]

Supplementary Information

Title: DNA methylation clocks for dogs and humans

Steve Horvath, Ake T. Lu[#], Amin Haghani[1#], Joseph A. Zoller, Caesar Z. Li, Andrea R. Lim, Robert T. Brooke, Ken Raj, Aitor Serres-Armero, Dayna L. Dreger, Jocelyn Plassais, Andrew N. Hogan, Elaine A. Ostrander

Corresponding authors: SH (shorvath@mednet.ucla.edu) EAO (eostrand@mail.nih.gov)

This file contains the following material

1) Supplementary Figures
2) Supplementary Note 1: References for dog breed lifespans.
3) Supplementary Note 2: Technical details surrounding the epigenetic clocks and R software code.
4) Supplementary Note 3: Details regarding the overlap analysis between dog EWAS and human GWAS.
5) Supplementary Note 4: CpG methylation influence on nearby gene transcription.
6) Supplementary Note 5: Quantification of CpG distal promoter/enhancer interactions.

Supplementary Figures

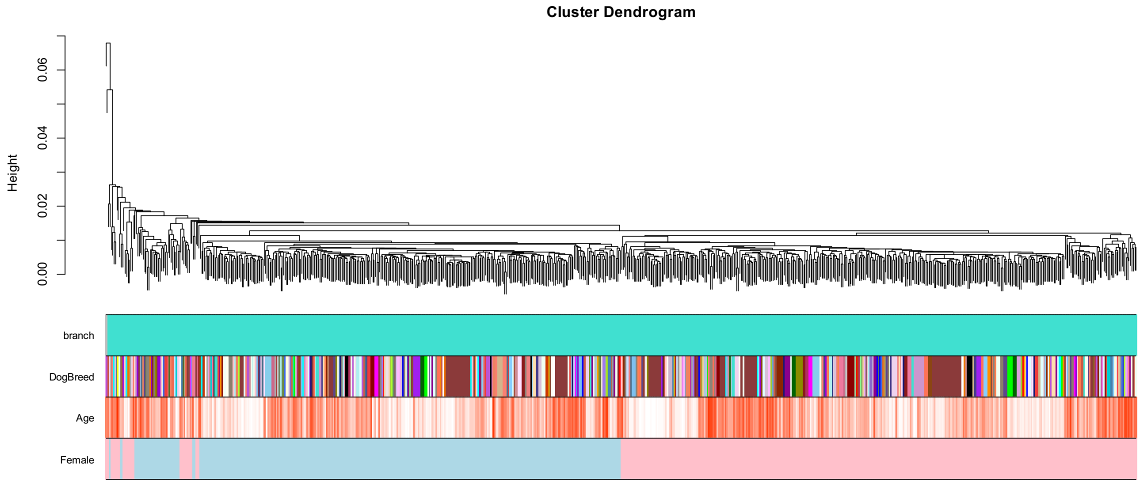

Supplementary Figure S1. Unsupervised hierarchical clustering reveals that canine methylation profiles cluster primarily by sex. Canine blood samples (n = 742 representing 93 breeds) cluster into a single group at a height cut-off of 0.04 (indicated by the 'branch' covariate), and separate into two groups primarily driven by sex rather than breed. Average linkage hierarchical clustering is based on the inter-array correlation. The dissimilarity measure is defined as one minus the Pearson correlation coefficient. For age covariate, white indicates low values and red indicates high values.

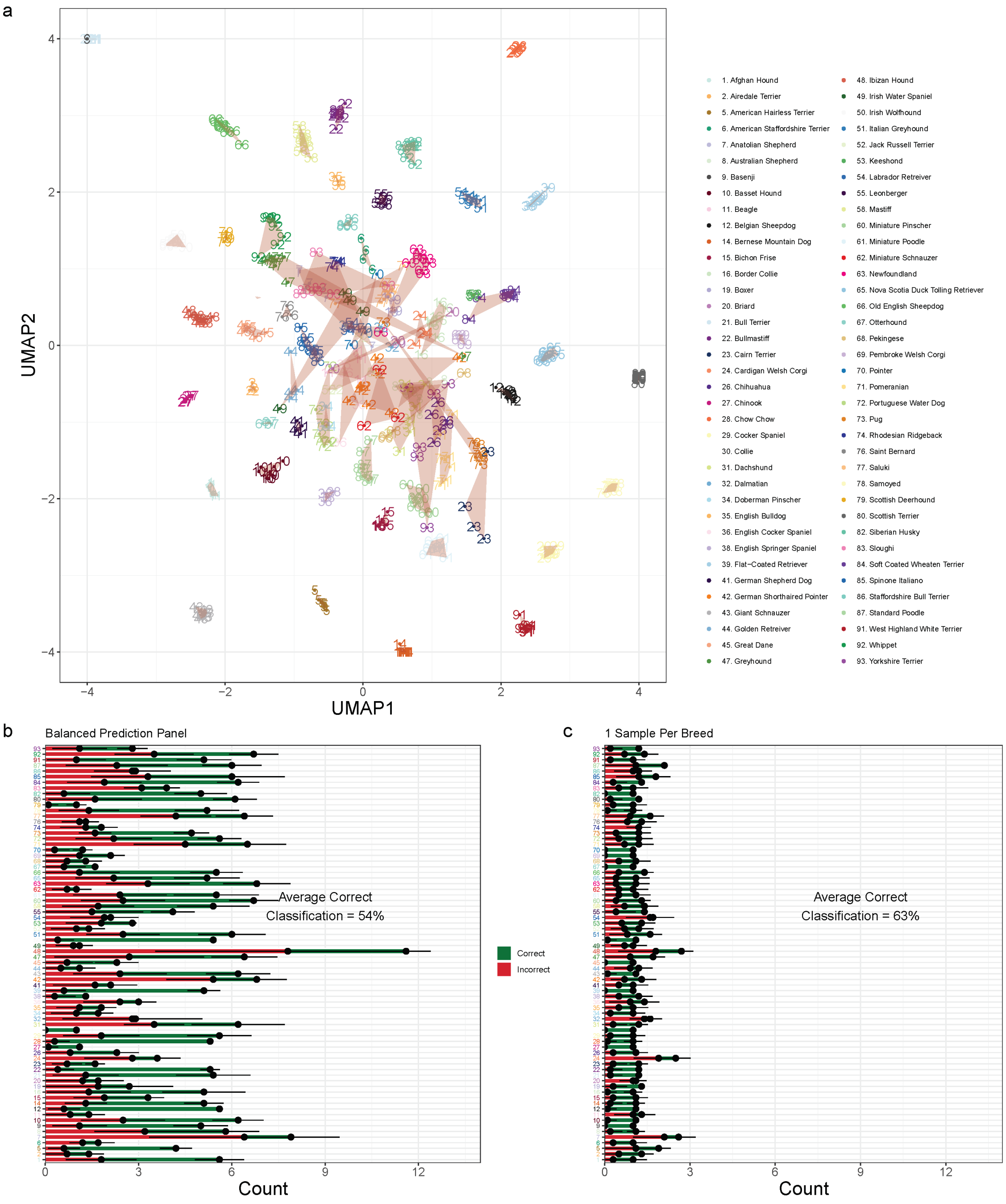

Supplementary Figure S2. High order principal component (PC) breed classification with autosomal methylation markers. PCs 1 through 7 were removed from the original data by projecting samples against the orthogonal PC space. Breeds with three or fewer samples were excluded from this analysis. **a**) Two-dimensional representation of the PC-reduced methylation dataset using UMAP (1). Samples belonging to the same breed are joined together by a semi-transparent polygon. **b, c**) Average number of correct and incorrect breed classifications using elastic net multinomial regression (α=0.5, λ=0.001) (2) across 10 sub-samplings of the PC-reduced panel. Error lines correspond to the average number of correct/incorrect classifications ± standard deviation. The classifier was trained on either **b**) approximately half the samples from every breed

while the remaining half was used for prediction, or **c**) all but one sample from every breed with the remaining sample used for prediction.

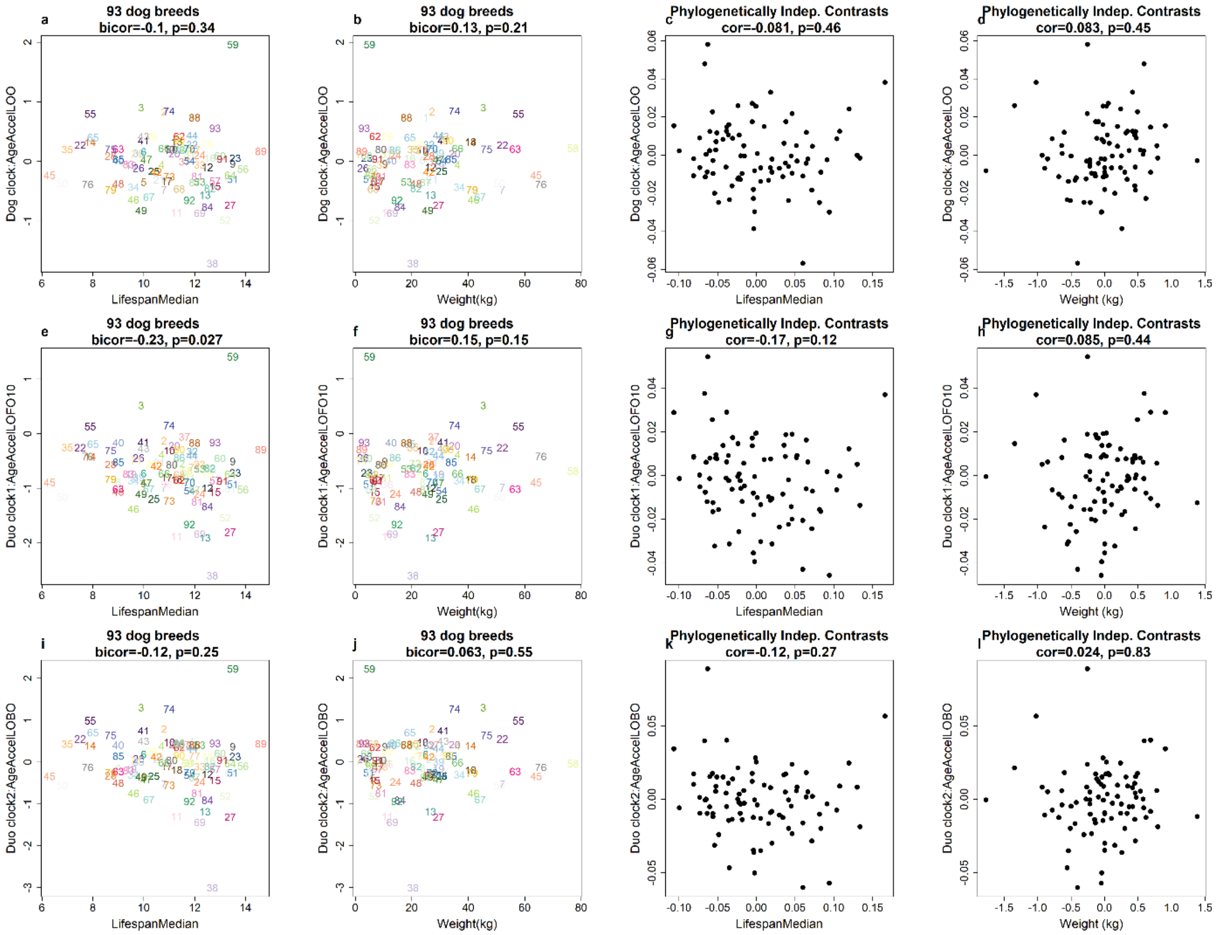

Supplementary Figure S3. Association of age epigenetic clocks with breed characteristics.

Association analysis for age acceleration measured by three clocks: **a-d**) pure dog epigenetic clock of age; **e-h**) human-dog clock for chronological age; and **i-l**) human-dog clock for relative age. All age acceleration measures are with respect to DNAm age estimates, in units of years. For each panel, we report the Pearson correlation estimate (cor) and corresponding Student's t-test p-value. Breeds index and colors are specified in the legend of ***SI Appendix*, Fig. S2**.

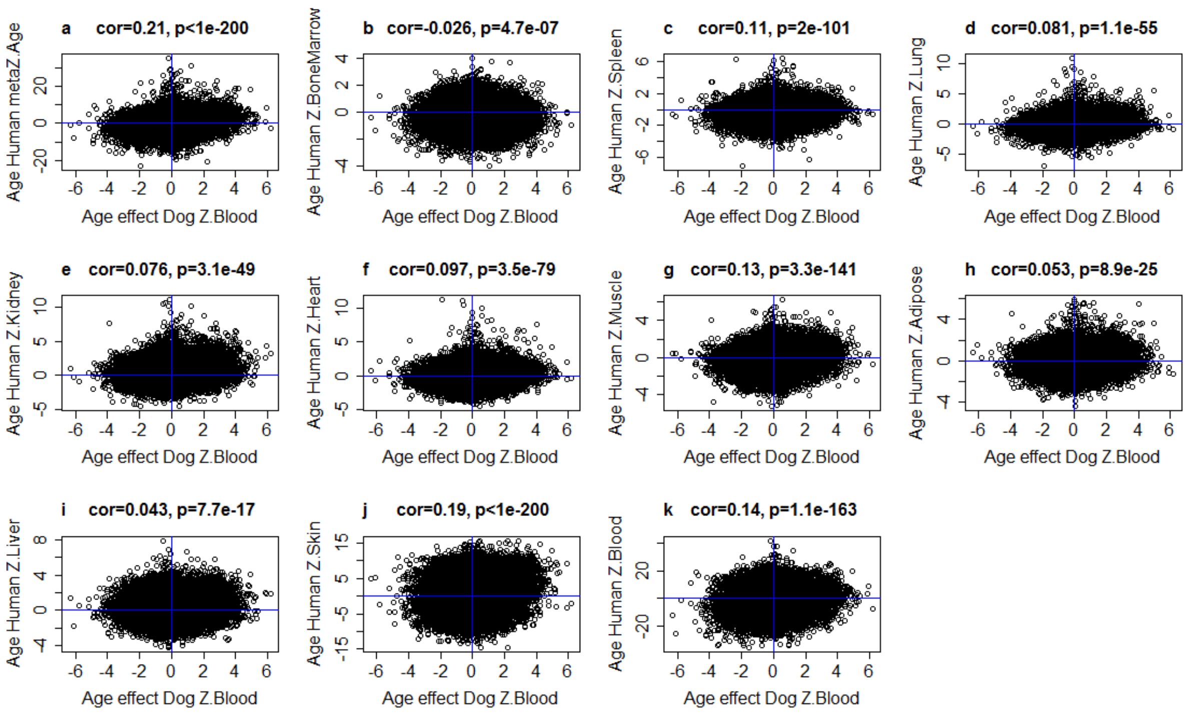

Supplementary Figure S4. **Age effects in methylation across tissues and species**. Correlation tests relating CpG methylation to chronological age across different dog and human tissues were compared using a Z statistic (Student's t-test statistic). **a**) Meta-analysis aggregating the age effect in all human tissues (Stouffer's method) versus dog blood. **b-k**) Individual human tissue age effects versus dog blood, including **b**) bone marrow, **c**) spleen, **d**) lung, **e**) kidney, **f**) heart, **g**) skeletal muscle, **h**) adipose, **i**) liver, **j**) skin, and **k**) blood. Each dot corresponds to a CpG on the mammalian array. Positive Z statistic values indicate a positive correlation between age and the CpG, *i.e.,* age-related gain of methylation. Z statistic values larger than 2 (or smaller than -2) correspond approximately to a two-sided correlation test $P < 0.05$.

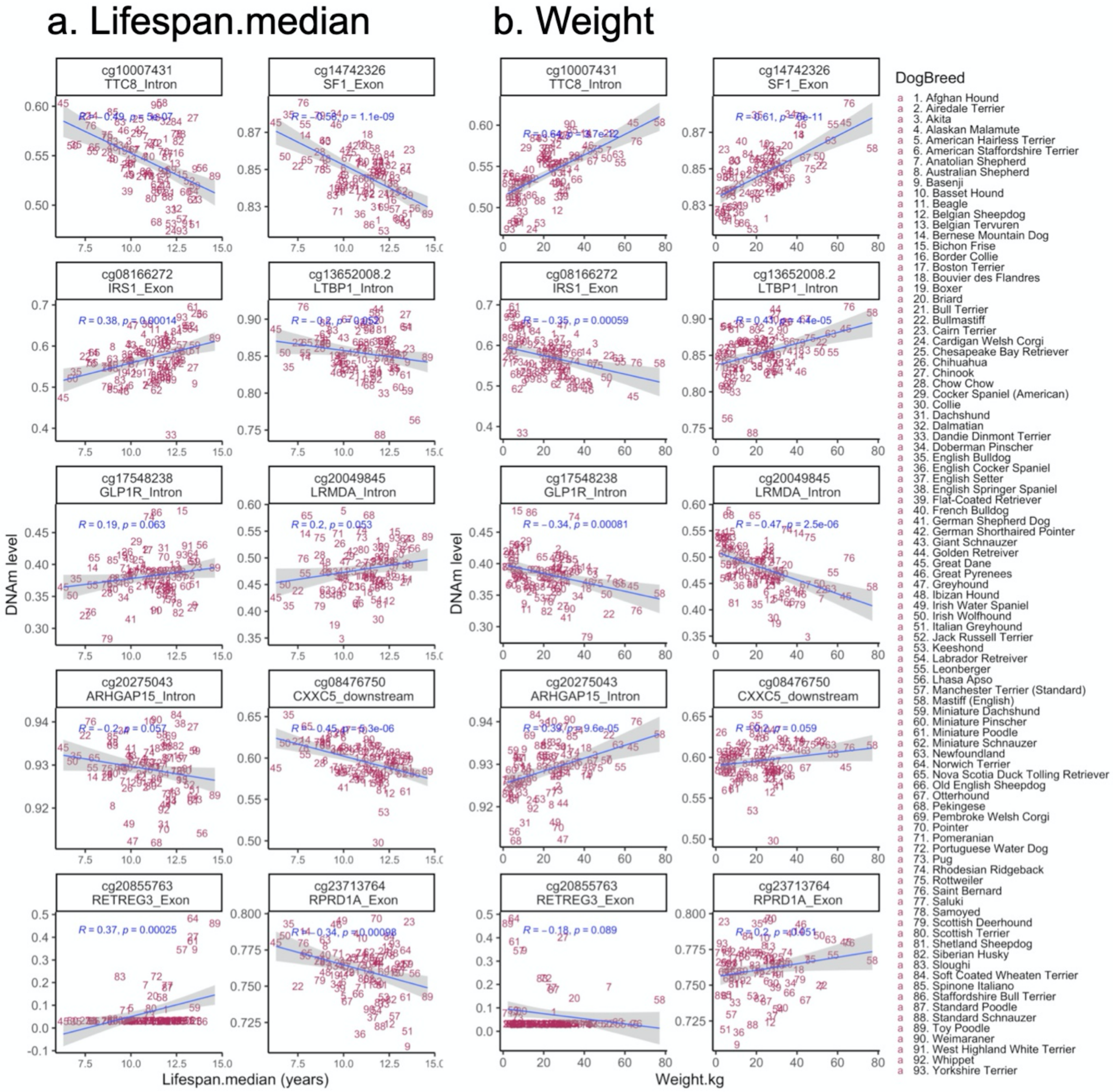

Supplementary Figure S5. Individual CpGs that relate to breed lifespan and weight. Scatterplots depicting the top 10 CpGs that relate to both lifespan (left) and weight (right) (see **Dataset S4**). Each panel reports the CpG and adjacent gene as well the Pearson correlation coefficient (R) and the p-value (in blue).

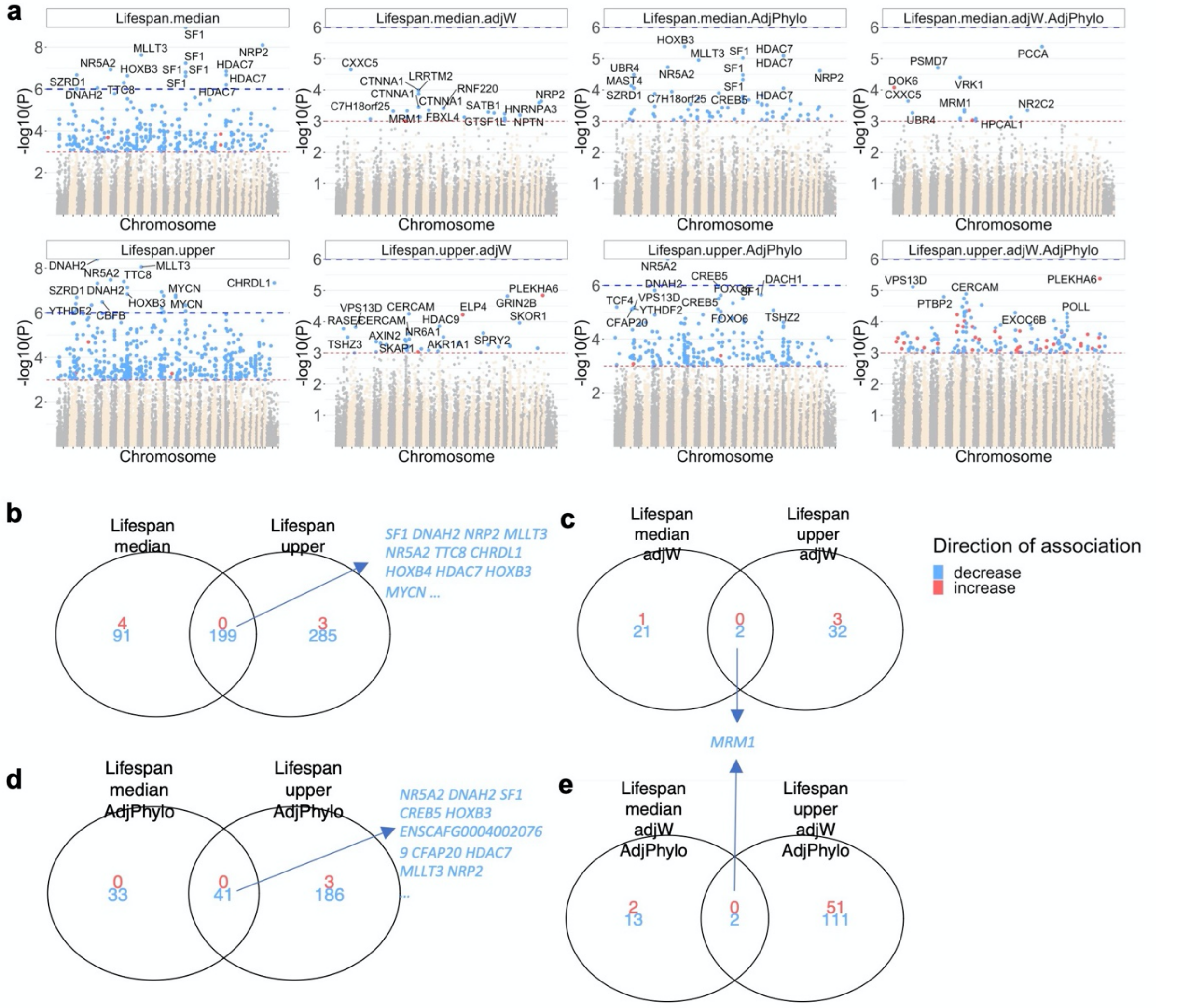

Supplementary Figure S6. EWAS of dog breed lifespan and DNA methylation. **a)** Manhattan plots of the EWAS results for two different lifespan measures, median lifespan and upper limit of the respective breed. The EWAS of lifespan was carried out using two approaches, covariate unadjusted or phylogenetic regression, and either unadjusted or adjusted for breed weight ('adjW'). The top 15 most significant CpGs are labeled according to their respective neighboring genes. Genome coordinates come from aligning the CpGs of the mammalian array to the genome assembly CanFam_GreatDane.UMICH_Zoey_3.1.100. Red dots correspond to a positive association between DNAm and each breed trait, whereas blue dots represent a negative correlation. The red dashed line indicates a significance threshold of $P < 10^{-3}$; the blue line indicates a Bonferroni-corrected significance level of $P < 1\text{x}10^{-6}$, which approximately corresponds to correcting for all probes on the mammalian array ($0.05 / 37492 = 1.3\text{x}10^{-6}$). No CpGs were significant at $P < 1\text{x}10^{-6}$ after adjusting for breed weight. **b-e)** Overlap of significant CpGs ($P < 10^{-3}$) between EWAS models of median and upper limit of breed lifespan, and the direction of the association between methylation and each trait.

Supplementary Note 1: References for the estimates of breed lifespan.

We used the following data sources for estimating the median lifespan and upper confidence interval of breed lifespan.

Adams, V. J., Evans, K. M., Sampson, J., & Wood, J. L. (2010). Methods and mortality results of a health survey of purebred dogs in the UK. *J Small Anim Pract*, *51*(10), 512-524. https://doi.org/10.1111/j.1748-5827.2010.00974.x

Afghan Hound, Airedale Terrier, Akita, Alaskan Malamute, Anatolian Shepherd, Australian Shepherd, Basenji, Basset Hound, Beagle, Belgian Sheepdog, Bernese Mountain Dog, Bichon Frise, Border Collie, Boston Terrier, Bouvier des Flandres, Boxer, Briard, Bull Terrier, Bullmastiff, Cairn Terrier, Cardigan Welsh Corgi, Chesapeake Bay Retriever, Chihuahua, Chow Chow Cocker Spaniel (American), Collie, Dachshund, Dalmatian, Dandie Dinmont Terrier, Doberman Pinscher, English Bulldog, English Cocker Spaniel, English Setter, English Springer Spaniel, Flat-Coated Retriever, French Bulldog, German Shorthaired Pointer, Giant Schnauzer, Golden Retriever, Great Dane, Great Pyrenees, Greyhound, Irish Water Spaniel, Irish Wolfhound, Italian Greyhound, Keeshond, Labrador Retriever, Leonberger, Lhasa Apso, Manchester Terrier (Standard), Mastiff (English), Miniature Pinscher, Miniature Poodle, Miniature Schnauzer, Newfoundland, Norwich Terrier, Nova Scotia Duck Tolling Retriever, Old English Sheepdog, Otterhound, Pekingese, Pembroke Welsh Corgi, Pointer, Pomeranian, Portuguese Water Dog, Pug, Rhodesian Ridgeback, Rottweiler, Saint Bernard, Saluki, Samoyed, Scottish Deerhound, Scottish Terrier, Shetland Sheepdog, Siberian Husky, Soft Coated Wheaten Terrier, Spinone Italiano, Staffordshire Bull Terrier, Standard Poodle, Standard Schnauzer, Toy Poodle, Weimaraner, West Highland White Terrier, Whippet, Yorkshire Terrier

American Chesapeake Club. (2005). *Chesapeake Bay Retriever Health Survey*. http://www.amchessieclub.org/survey/index.html

Chesapeake Bay Retriever

Bell, L., & Hesketh, S. (2021). Mastiff breed mortality: A study of owner experience, dog age and longevity. *Vet Anim Sci*, *13*, 100194. https://doi.org/10.1016/j.vas.2021.100194

Bullmastiff, Great Dane, Mastiff (English)

Erickson, M., Freiheit, D., Gaburri, C., Lapp, A., Loland, K., Redfern, M., & Spaeth, A. (2009). *Old English Sheepdog Club Of America 2009 Breed Health Survey Report*. https://oldenglishsheepdogclubofamerica.org/wp-content/uploads/2020/06/OESHealthSurveyReport2009.pdf

Old English Sheepdog

Glickman, L., Glickman, N., & Thorpe, R. (1999). *The Golden Retriever Club of America National Health Survey 1998-1999*. https://grca.org/wp-content/uploads/2015/08/healthsurvey.pdf

Golden Retriever

Grayson, J. K. (2007). *West Highland White Terrier Health Survey Final Report*. https://westiefoundation.org/files/galleries/2005_westie_survey_results.pdf
West Highland White Terrier

Klopfenstein, M., Howard, J., Rossetti, M., & Geissbuhler, U. (2016). Life expectancy and causes of death in Bernese mountain dogs in Switzerland. *BMC Vet Res*, *12*(1), 153. https://doi.org/10.1186/s12917-016-0782-9
Bernese Mountain Dog

Leroy, G., Phocas, F., Hedan, B., Verrier, E., & Rognon, X. (2015). Inbreeding impact on litter size and survival in selected canine breeds. *Vet J*, *203*(1), 74-78. https://doi.org/10.1016/j.tvjl.2014.11.008
Basset Hound, Bernese Mountain Dog, Cairn Terrier, German Shepherd Dog, Leonberger, West Highland White Terrier

Lewis, T. W., Wiles, B. M., Llewellyn-Zaidi, A. M., Evans, K. M., & O'Neill, D. G. (2018). Longevity and mortality in Kennel Club registered dog breeds in the UK in 2014. *Canine Genet Epidemiol*, *5*, 10. https://doi.org/10.1186/s40575-018-0066-8
Bernese Mountain Dog, Border Collie, Boxer, Dalmatian, Doberman Pinscher, English Cocker Spaniel, Flat-Coated Retriever, German Shepherd Dog, Golden Retriever, Labrador Retriever, Miniature Schnauzer, Newfoundland, Pointer, Rottweiler, Shetland Sheepdog, Staffordshire Bull Terrier, Weimaraner, West Highland White Terrier, Whippet

McGreevy, P. D., Wilson, B. J., Mansfield, C. S., Brodbelt, D. C., Church, D. B., Dhand, N., Soares Magalhaes, R. J., & O'Neill, D. G. (2018). Labrador retrievers under primary veterinary care in the UK: demography, mortality and disorders. *Canine Genet Epidemiol*, *5*, 8. https://doi.org/10.1186/s40575-018-0064-x
Labrador Retriever

Moreland, S. (2008). *Life Span Study of the Irish Water Spaniel in America*. https://www.iwsca.org/Health/2008%20Lifespan%20Study.pdf
Irish Water Spaniel

O'Neill, D. G., Ballantyne, Z. F., Hendricks, A., Church, D. B., Brodbelt, D. C., & Pegram, C. (2019). West Highland White Terriers under primary veterinary care in the UK in 2016: demography, mortality and disorders. *Canine Genet Epidemiol*, *6*, 7. https://doi.org/10.1186/s40575-019-0075-2
West Highland White Terrier

O'Neill, D. G., Butcher, C., Church, D. B., Brodbelt, D. C., & Gough, A. G. (2019). Miniature Schnauzers under primary veterinary care in the UK in 2013: demography, mortality and disorders. *Canine Genet Epidemiol*, *6*, 1. https://doi.org/10.1186/s40575-019-0069-0
Miniature Schnauzer

O'Neill, D. G., Church, D. B., McGreevy, P. D., Thomson, P. C., & Brodbelt, D. C. (2013). Longevity and mortality of owned dogs in England. *Vet J*, *198*(3), 638-643. https://doi.org/10.1016/j.tvjl.2013.09.020
Bichon Frise, Border Collie, Boxer, Bull Terrier, Cairn Terrier, Chihuahua, Collie, Dalmatian, Doberman Pinscher, English Bulldog,

English Cocker Spaniel, English Springer Spaniel, German Shepherd Dog Golden Retriever, Great Dane, Greyhound, Jack Russell Terrier, Labrador Retriever, Lhasa Apso, Mastiff (English), Miniature Dachshund, Miniature Poodle, Rottweiler, Scottish Terrier, Shetland Sheepdog, Staffordshire Bull Terrier, Weimaraner, West Highland White Terrier, Yorkshire Terrier

O'Neill, D. G., Coulson, N. R., Church, D. B., & Brodbelt, D. C. (2017). Demography and disorders of German Shepherd Dogs under primary veterinary care in the UK. *Canine Genet Epidemiol*, *4*, 7. https://doi.org/10.1186/s40575-017-0046-4
German Shepherd Dog

O'Neill, D. G., Rooney, N. J., Brock, C., Church, D. B., Brodbelt, D. C., & Pegram, C. (2019). Greyhounds under general veterinary care in the UK during 2016: demography and common disorders. *Canine Genet Epidemiol*, *6*, 4. https://doi.org/10.1186/s40575-019-0072-5
Greyhound

O'Neill, D. G., Seah, W. Y., Church, D. B., & Brodbelt, D. C. (2017). Rottweilers under primary veterinary care in the UK: demography, mortality and disorders. *Canine Genet Epidemiol*, *4*, 13. https://doi.org/10.1186/s40575-017-0051-7
Rottweiler

O'Neill, D. G., Skipper, A. M., Kadhim, J., Church, D. B., Brodbelt, D. C., & Packer, R. M. A. (2019). Disorders of Bulldogs under primary veterinary care in the UK in 2013. *PLoS One*, *14*(6), e0217928. https://doi.org/10.1371/journal.pone.0217928
English Bulldog

Proschowsky, H. F., Rugbjerg, H., & Ersboll, A. K. (2003). Mortality of purebred and mixed-breed dogs in Denmark. *Prev Vet Med*, *58*(1-2), 63-74. https://doi.org/10.1016/s0167-5877(03)00010-2
Beagle, Bernese Mountain Dog, Boxer, Chow Chow, Collie, Dachshund, Doberman Pinscher, English Cocker Spaniel, Flat-Coated Retriever, German Shepherd Dog, German Shorthaired Pointer, Golden Retriever, Labrador Retriever, Miniature Poodle, Newfoundland, Rottweiler, Saint Bernard, Shetland Sheepdog, Standard Poodle

Stivason, K. (2020). *The Mastiff Club of America Longevity Study*. Retrieved October 25, 2021 from https://www.mastiff.org/longevity-study/
English Mastiff

The American Boxer Club. (2012). *The American Boxer Club Health Survey Report*. https://americanboxerclub.org/PDF/ABC-Health-Report-Aug-2012.pdf
Boxer

The Dalmatian Club of America. (2003, November 19, 2003). *The Dalmatian Club of America Health Survey Results*. Retrieved October 25, 2021 from https://www.thedca.org/healthcon.html
Dalmatian

The Kennel Club. (2004). *2004 Pedigree Dog Health Survey*. https://www.thekennelclub.org.uk/health-and-dog-care/what-we-do-for-dog-health/supporting-scientific-research/kennel-club-health-research/2004-pedigree-dog-health-survey/.

Lhasa Apso, Standard Manchester Terrier, Whippet, Keeshond, Miniature Schnauzer, Pekingese, Basset Hound, Pug, Greyhound, Saint Bernard, English Bulldog

Toller Health Coalition. (2002). *Nova Scotia Duck Tolling Retriever Health Survey*. Retrieved October 25, 2021 from https://web.archive.org/web/20130709021639/http://www.toller.ca/tollerhealth/SurveySummary.html#

Nova Scotia Duck Tolling Retriever

References:
(3-26)

Reference Type: Report
Author: American Chesapeake Club
Year: 2005
Title: American Chesapeake Club Health Survey
Date: May 2005
Short Title: American Chesapeake Club Health Survey
internal-pdf://3829176425/ACCHealthSurveyReport2005.pdf

Supplementary Note 2: Technical details surrounding epigenetic clocks

Statistical methods used for building the clocks

The epigenetic clocks were used by employing a single elastic net regression model analysis (R function glmnet). We use used Leave-one-out analysis (LOO) using a single lambda value. We chose the following parameters for the glmnet R function (Alpha: 0.5, CV Fold: 10, Lambda choice for Clock: 1 standard error above minimum CV-MSE).

Covariates and coefficient values of the dog clocks

The coefficient values of the clocks are specified in **Dataset S8**.

1) The dog clock for blood is based on 52 CpGs whose coefficient values are specified in the column "Coef.DogBlood". Age transformation=identity, i.e. F(Age)=Age
2) The human dog clock for chronological age is based on 561 CpGs whose coefficient values are specified in the column "Coef.HumanDogLogLinearAge". Age transformation=log-linear described below.
3) The human dog clock for relative age is based on 497 CpGs whose coefficient values are specified in the column "Coef.HumanDogRelativeAge". Age transformation: relative age. i.e. F(Age)=Age/maxLifespan. Max lifespan for dogs is 24 years. Human max lifespan =122.5 years.
4) The clock for estimating Average Time To Death (Coef.DogAverageTimeToDeath) is based on 340 CpGs. No transformation of the dependent variable.

General description of age transformation

The human-dog clocks for chronological age used log linear transformations that are similar to those employed for the HUMAN pan tissue (Horvath 2013) (27).

An elastic net regression model (implemented in the glmnet R function) was used to regress a transformed version of age on the beta values in the training data. The glmnet function requires the user to specify two parameters (alpha and beta). Since I used an elastic net predictor, alpha was set to 0.5. But the lambda value of was chosen by applying a 10 fold cross validation to the training data (via the R function cv.glmnet).

The elastic net regression results in a linear regression model whose coefficients b_0, b_1, . . . , relate to transformed age as follows

$F(\text{chronological age})=b_0+b_1 CpG_1+ \ldots +b_p CpG_p+\text{error}$

Note that the intercept term is denoted by b_0. The coefficient values can be found in **Dataset S8**. Based on the coefficient values from the regression model, DNAmAge is estimated as follows

$DNAm\text{Age}=F^{-1}(b_0+b_1 CpG_1+ \ldots +b_p CpG_p)$

, where $F^{-1}(y)$ denotes the mathematical inverse of the function F(.). Thus, the regression model can be used to predict to transformed age value by simply plugging the beta values of the selected CpGs into the formula.

Defining Properties of the log linear transformation

As indicated by its name, the "log-linear" function, has a logarithmic dependence on age before the average age of sexual maturity (of the species) and a linear dependence after Age at Sexual Maturity (of the species). For the human-dog clocks we used the following averages at sexual maturity (in units of years): 13.5 years for humans and 1.83287671232877 years for dogs.

We used a piecewise transformation, parameterized by Age of Sexual Maturity (A).

The transformation is F(x), given by

$$F(x) = g\left(\frac{x+1.5}{A+1.5}\right) \text{ where } g(t) = \begin{cases} \log(t), & for\ 0 \leq t \leq 1 \\ t-1, & for\ 1 \leq t \end{cases}$$

Explicitly, F(x) is given by

$$F(x) = \begin{cases} \log\left(\frac{x+1.5}{A+1.5}\right), & for\ 0 \leq x \leq A \\ \frac{x-A}{A+1.5}, & for\ A \leq x \end{cases}$$

In order to use this transformation to predict Age on *new samples*, one needs to use the *inverse* transformation, $F^{-1}(y)$, given by

$$F^{-1}(y) = \begin{cases} (A+1.5) * \exp(y) - 1.5, & for\ y \leq 0 \\ (A+1.5)y + A, & for\ y \geq 0 \end{cases}$$

For predicting age, apply the inverse transformation to coefficient-weighted sum. That is,

$$DNAmAge = F^{-1}(x * \beta)$$

where β is the vector of coefficients and x is the vector of methylation values, with an intercept term.

R Implementation of the log linear transformation

```
### Applies the log linear transformation to the input vector x, i.e. to Age
F= Vectorize(function(x, maturity, ...) {
  if (is.na(x) | is.na(maturity)) {return(NA)}
  k <- 1.5
  y <- 0
  if (x < maturity) {y = log((x+k)/(maturity+k))}
  else {y = (x-maturity)/(maturity+k)}
```

```
  return(y)
})
### Inverse log linear transformation
F.inverse= Vectorize(function(y, maturity, ...) {
  if (is.na(y) | is.na(maturity)) {return(NA)}

  k <- 1.5
  x <- 0
  if (y < 0) {x = (maturity+k)*exp(y)-k}
  else {x = (maturity+k)*y+maturity}
  return(x)
})
```

The DNAm Age estimate is estimated in two steps.

First, one forms a weighted linear combination of the CpGs whose details can be found in **Dataset S8**.

The table reports the probe identifier (cg number) used in the custom Infinium array (HorvathMammalMethylChip40). The weights used in this linear combination are specified in the respective column entitled "Coef.".

The formula assumes that the DNA methylation data measure "beta" values but the formula could be adapted to other ways of generating DNA methylation data.

Pseudo R code

```
# R function for multivariate regression model
multivariatePredictorCoef=function(dat0, datCOEF,imputeValues=FALSE) {
datout=data.frame(matrix(NA,nrow=dim(dat0)[[2]]-1,ncol=dim(datCOEF)[[2]]-1 ))
match1=match(datCOEF[-1,1],dat0[,1] )
if (   sum(!is.na(match1))==0 ) stop("Input error. The first column of dat0 does not contain CpG identifiers (cg numbers).")
dat1=dat0[match1,]
row.names1=as.character(dat1[,1])
dat1=dat1[,-1]
if (imputeValues ){dat1=impute.knn(data=as.matrix(dat1) ,k = 10)[[1]]}
for (i in 1:dim(dat1)[[2]] ){ for (j in 2:dim(as.matrix(datCOEF))[[2]] ){
```

```
datout[i,j-1]=sum(dat1[,i]* datCOEF[-1,j],na.rm=TRUE)+ datCOEF[1,j]}}
colnames(datout)=colnames(datCOEF)[-1]
rownames(datout)=colnames(dat0)[-1]
datout=data.frame(SampleID= colnames(dat0)[-1],datout)
datout
} # end of function

# read in supplementary table
datCoef=read.csv("TableS.csv")

The first columns should read as follows
names(datCoef)
[1] "var"
[2] "Coef.DogBlood"
[3] "Coef.HumanDogAgeLogLinear"
[4] "Coef.HumanDogRelativeAge"
[5] "Coef.DogAverageTimeToDeath"

# Restrict attention to the first 5 columns
datCoef=datCoef[,c(1:5)]

# assume the first column of dat0 contains the CpG identifiers
match1=match(datCoef[-1,1],dat0[,1] )
missingProbes= as.character(datCoef[-1,1] )[is.na(match1)]

dat1=dat0[match1,]
# data frame with predicted values.
datPredictions=multivariatePredictorCoef(dat1,datCOEF=datCoef,imputeVa
lues=FALSE)

#let's relabel the columns by replacing "Coef" with "DNAm" since the
columns contain estimates of age or relative age instead of
coefficient values
```

```
colnames(datPredictions)=gsub(pattern="Coef", replacement="DNAm", x=colnames(datPredictions))


# We need to transform the human dog clock for chronological age using the inverse of the log linear #transformation. For dogs, the age at sexual maturity has to be set to 1.83287671232877 years.

datPredictions$DNAm.HumanDogAgeLogLinear= F.inverse(datPredictions$DNAm.HumanDogAgeLogLinear, maturity=1.83287671232877)

#The data frame "datPredictions" contains the age estimates in units of years and relative age estimates.
```

Supplementary Note 3: Details regarding the overlap analysis between dog EWAS and human GWAS.

We related our EWAS results in dogs with a broad category of human GWAS studies: anthropometric traits, behavioral phenotypes, cognitive related traits, fetal growth traits, inflammatory diseases, lipid panel outcomes, metabolic outcomes and diseases, neurodegenerative and neuropsychiatric disorders, longevity, reproductive aging and other age related phenotypes including DNA methylation based biomarkers. All GWAS results are based on meta-analysis across large-scale human studies. For instance, GWAS of anthropometric traits involved more than 200k individuals from multiple ethnic groups, conducted by the GIANT consortium, https://portals.broadinstitute.org/collaboration/giant/index.php/GIANT_consortium. We used the MAGENTA software(28) to define gene level p-values for the human genome wide association studies (GWAS) results from a total of 70 large-scale GWAS studies (29-47). For each GWAS, we focused on the top 2.5% genes for downstream enrichment analysis. Other thresholds would lead to qualitatively similar results. The EWAS-GWAS enrichment analysis was based on genomic-region hypergeometric analysis as described in ***Materials and Methods***. The GWAS summary datasets were downloaded from OpenGWAS (48-50) (https://gwas.mrcieu.ac.uk/) or obtained from the corresponding study groups. Citations to the respective scientific papers are provided below in **Table SN1**.

Index	Hg	Category	Trait	Ethnicity	Sex	PMID
1	hg19	Neurodegenerative disorder	Age-related Macular degeneration (AMD)	EUR+ASN	All	23455636
2	hg19	Neurodegenerative disorder	AMD Geographic Atrophy	EUR+ASN	All	23455636
3	hg19	Neurodegenerative disorder	AMD Neovascular	EUR+ASN	All	23455636
4	hg19	Neurodegenerative disorder	Alzheimer's disease	EUR	All	24162737
5	hg18	Longevity	Longevity > 90	EUR	All	24688116
6	hg18	Longevity	Longevity > 85	EUR	All	24688116
7	hg19	Neurodegenerative disorder	Parkinson's disease	EUR	All	19915575
8	hg19	Neuropsychiatric disorder	Schizophrenia	All	All	25056061
9	hg19	Inflammatory diseases	IBD	EUR	All	26192919
10	hg19	Inflammatory diseases	IBD Crohn's disease	EUR	All	26192919

11	hg19	Inflammatory diseases	IBD Ulcerative colitis	EUR	All	26192919
12	hg18	Neuropsychiatric disorder	Bipolar disorder	All	All	21926972
13	hg18	Neuropsychiatric disorder	ADHD	All	All	20732625
14	hg18	Neuropsychiatric disorder	Major depression disorder	EUR	All	22472876
15	hg18	Metabolic outcomes and diseases	Type 2 diabetes	EUR	All	22885922
16	hg18	Metabolic outcomes and diseases	Fasting glucose	EUR	All	22581228
17	hg18	Metabolic outcomes and diseases	Fasting insulin	EUR	All	22581228
18	hg18	GIANT Body fat distribution	Hip AllAncestries	ALL	M&F	25673412
19	hg18	GIANT Body fat distribution	Hip EUR	EUR	M&F	25673412
20	hg18	GIANT Body fat distribution	Hip AllAncestries(Males)	ALL	M	25673412
21	hg18	GIANT Body fat distribution	Hip EUR (Males)	EUR	M	25673412
22	hg18	GIANT Body fat distribution	Hip AllAncestries(Females)	ALL	F	25673412
23	hg18	GIANT Body fat distribution	Hip EUR (Females)	EUR	F	25673412
30	hg18	GIANT Body fat distribution	Waist circumference AllAncestries	ALL	M&F	25673412
31	hg18	GIANT Body fat distribution	Waist circumference EUR	EUR	M&F	25673412
32	hg18	GIANT Body fat distribution	Waist circumference AllAncestries(Males)	ALL	M	25673412
33	hg18	GIANT Body fat distribution	Waist circumference EUR (Males)	EUR	M	25673412
34	hg18	GIANT Body fat distribution	Waist circumference AllAncestries(Females)	ALL	F	25673412
35	hg18	GIANT Body fat distribution	Waist circumference EUR (Females)	EUR	F	25673412

42	hg18	GIANT Body fat distribution	Waist to hip ratio AllAncestries	ALL	M&F	25673412
43	hg18	GIANT Body fat distribution	Waist to hip ratio EUR	EUR	M&F	25673412
44	hg18	GIANT Body fat distribution	Waist to hip ratio AllAncestries(Males)	ALL	M	25673412
45	hg18	GIANT Body fat distribution	Waist to hip ratio EUR (Males)	EUR	M	25673412
46	hg18	GIANT Body fat distribution	Waist to hip ratio AllAncestries(Females)	ALL	F	25673412
47	hg18	GIANT Body fat distribution	Waist to hip ratio EUR (Females)	EUR	F	25673412
54	hg18	GIANT BMI & Height	BMI	EUR	All	25673413
55	hg18	GIANT BMI & Height	Height	EUR	All	20881960
56	hg19	Neurodegenerative disorder	Frontotemporal dementia	EUR	All	24943344
57	hg19	Neurodegenerative disorder	FTD Behavioral variant	EUR	All	24943344
58	hg19	Neurodegenerative disorder	FTD with motor neuron disease	EUR	All	24943344
59	hg19	Neurodegenerative disorder	FTD progressive non-fluent aphasia	EUR	All	24943344
60	hg19	Neurodegenerative disorder	FTD semantic dementia	EUR	All	24943344
61	hg19	Neurodegenerative disorder	Huntington's disease age onset	EUR	All	26232222
62	hg19	Behavioral phenotype	Educational attainment	EUR	All	27225129
63	hg19	Behavioral phenotype	Educational attainment (Males)	EUR	All	27225129
64	hg19	Behavioral phenotype	Educational attainment (Females)	EUR	All	27225129
65	hg18	Reproductive aging	Age at menarche	EUR	All	25231870
66	hg18	Reproductive aging	Age at menopause	EUR	All	26414677

67	hg18	Lipid panel outcomes	HDL		All	24097068
68	hg18	Lipid panel outcomes	LDL		All	24097068
69	hg18	Lipid panel outcomes	Total cholesterol		All	24097068
70	hg18	Lipid panel outcomes	Triglyceride		All	24097068
71	hg18	Reproductive aging	Leukocyte telomere length	EUR	All	23535734
72	hg19	DNAm biomarkers	AgeAccelGrim EUR	EUR	All	34187551
73	hg19	DNAm biomarkers	DNAmGranAdjustedAge EUR	EUR	All	34187551
74	hg19	DNAm biomarkers	AgeAccelHannum EUR	EUR	All	34187551
75	hg19	DNAm biomarkers	DNAmPAI1AdjAge EUR	EUR	All	34187551
76	hg19	DNAm biomarkers	IEAA EUR	EUR	All	34187551
77	hg19	DNAm biomarkers	AgeaccelPhenoAge EUR	EUR	All	34187551
78	hg19	DNAm biomarkers	AgeAccelGrim AFR	AFR	All	34187551
79	hg19	DNAm biomarkers	DNAmGranAdjustedAge AFR	AFR	All	34187551
80	hg19	DNAm biomarkers	AgeAccelHannum AFR	AFR	All	34187551
81	hg19	DNAm biomarkers	DNAmPAI1AdjAge AFR	AFR	All	34187551
82	hg19	DNAm biomarkers	IEAA AFR	AFR	All	34187551
83	hg19	DNAm biomarkers	AgeaccelPhenoAge AFR	AFR	All	34187551
84	hg19	DNAm biomarkers	AgeAccelGrim All	EUR+AFR	All	34187551
85	hg19	DNAm biomarkers	DNAmGranAdjustedAge All	EUR+AFR	All	34187551
86	hg19	DNAm biomarkers	AgeAccelHannum All	EUR+AFR	All	34187551
87	hg19	DNAm biomarkers	DNAmPAI1AdjAge All	EUR+AFR	All	34187551
88	hg19	DNAm biomarkers	IEAA All	EUR+AFR	All	34187551
89	hg19	DNAm biomarkers	AgeAccelPhenoAge All	EUR+AFR	All	34187551
90	hg19	Longevity	Father's attained age	EUR	All	29227965
91	hg19	Longevity	Mother's attained age	EUR	All	29227965
92	hg19	Longevity	Parental attained age	EUR	All	29227965

93	hg19	Age related phenotype	Atrial fibrillation	EUR	All	30061737
94	hg19	Neurodegenerative disorder	Alzheimer's disease	EUR	All	30617256
95	hg19	Cognitive related	Intelligence	EUR	All	29942086
96	hg19	Reproductive aging	AgeAtMenarche	EUR	All	28436984
97	hg19	Neurodegenerative disorder	Huntington's disease motor progression	EUR	All	28642124
98	hg19	Fetal growth	Birth length	EUR	All	34282336
99	hg19	Fetal growth	Infant Ponderal index	EUR	All	34282336
100	hg19	Fetal growth	Birth weight	EUR	All	34282336
101	hg19	Fetal growth	Birth weight fatherGenome	EUR	All	34282336
102	hg19	Fetal growth	Birth weight motherGenome	EUR	All	34282336
EUR: Europeans; AFR: Africans; ASN: Asians.						

Supplementary Notes Table SN1 – *Compilation of all GWAS results used for EWAS comparison.*

Supplementary Note 4: CpG methylation influence on nearby gene transcription.

We leveraged published canine RNA-seq and ChIP-seq data (**See Table SN2 below**) to understand the extent to which CpGs associated with age, lifespan and weight influence the transcriptional activity of nearby genes.

Data	Tissue	N samples	Study Accession/Publication
**RNA-seq**	Whole Blood	12	PRJNA790829
**RNA-seq**	Testes	9	PRJEB17926
**RNA-Seq**	Skin	9	PRJNA338147, PRJNA327075, PRJEB14109
**RNA-Seq**	Brain	13	PRJNA396033, PRJEB17926, PRJNA157897, PRJNA327075, PRJNA276284, PRJEB4668
**ChIP-seq H3K4m3**	Whole Blood	7	PRJNA687522
**ChIP-seq H3K4m1**	Whole Blood	7	PRJNA687522

Supplementary Notes Table SN2 *– Description of the multi-omics data used to assess transcription.*

The RNA-seq data was mapped against the CanFam3.1 reference gene annotation and processed according to the GTEx TOPMed pipeline, as detailed in (51). As a QC metric, we observed clustering by RNA-seq tissue regardless of study or coverage and found concordance between the transcription levels derived in our analysis, those obtained from the original publications and the human GTEx atlas (human-dog transcription level correlations for 26,204 genes: ρ_{blood}=0.78, ρ_{skin}=0.83, ρ_{testes}=0.77, ρ_{brain}=0.82). The ChIP-seq data was not modified, as it was already processed (52) and mapped to the CanFam3.1 genome build.

In concordance with their enrichment in polycomb repressive complex 2 (PRC2) binding signals, the genes assigned to age-associated CpGs were found to be more often untranscribed in all adult tissues compared to the methylation array transcriptional background (Fisher exact test P = 2.2x10-16 and (**See Table SN3 below**). Genes assigned to lifespan and weight CpGs had, on average, higher expression levels than the array background across all tissues (Wilcoxon rank sum test P = 9.5x10-15).

Symbol	Class	Blood1	Blood2	Testes1	Testes2	Skin1	Skin2	Brain1	Brain2
ONECUT2	Age	0.00	0.00	0.00	0.00	0.00	0.00	0.00	0.23

NR2F2	Age	0.00	0.00	2.52	3.66	2.27	2.67	0.00	2.82
KLHL14	Age	0.00	0.67	0.27	0.00	0.00	0.00	0.00	0.03
HNF1B	Age	0.00	0.00	2.17	0.00	0.00	0.00	0.00	0.00
ZNF521	Age	0.00	0.00	0.50	1.72	1.84	1.68	1.43	2.15
HCN1	Age	0.00	0.00	0.00	0.00	0.00	0.00	0.10	2.30
SGIP1	Age	0.00	0.00	1.09	1.01	0.00	1.02	1.25	3.76
SEZ6	Age	0.00	0.00	1.73	0.00	0.00	0.00	1.62	3.44
SEMA6B	Age	2.66	1.97	0.88	1.92	0.00	0.94	3.73	3.60
DPYD	Age	4.67	5.67	2.10	2.69	3.65	2.85	0.54	3.67
BARHL2	Age	0.00	0.00	0.24	1.46	0.00	0.00	0.00	0.00
LHX2	Age	0.00	0.00	0.00	2.23	3.86	4.88	3.06	2.76
ZFHX3	Age	0.00	0.00	1.15	0.05	0.00	0.40	0.00	2.21
FOXB1	Age	0.00	0.00	0.00	0.00	0.00	0.00	0.00	0.00
NEUROG2	Age	0.00	0.00	0.00	0.00	0.00	0.00	0.00	0.00
LBX1	Age	0.00	0.00	0.00	0.00	0.72	0.00	0.00	0.00
ANKRD52	divergent	2.10	2.64	4.13	1.86	1.06	1.97	0.00	3.84
TCF4	divergent	1.47	1.88	2.41	0.73	1.79	1.84	2.72	3.47
FKBP5	divergent	2.35	3.79	3.62	3.56	3.20	3.71	1.75	3.96
FBXL20	divergent	2.63	3.31	3.27	3.91	3.08	3.51	2.55	3.12
PEX14	divergent	2.99	2.79	3.55	4.40	3.48	4.05	4.57	3.87
HOXB5	divergent	0.00	0.00	0.00	0.00	0.00	0.01	0.00	0.00
ZNF609	divergent	1.63	1.79	3.39	1.53	1.87	2.38	0.17	3.08
OGT	divergent	4.75	4.98	4.11	3.43	3.94	4.95	1.53	5.07
CCDC182	divergent	0.00	0.00	2.97	0.00	0.00	0.00	0.00	0.00
TTC8	divergent	0.00	0.00	3.56	3.80	2.21	2.04	2.85	4.27
SMAD2	divergent	3.98	3.89	2.91	3.60	3.70	3.73	1.87	3.57
BCL11B	divergent	0.17	0.00	0.00	0.00	0.00	0.00	0.00	0.00
PSD2	lifespanOnly	1.04	1.68	3.09	2.88	2.80	2.80	3.83	3.89
ASXL1	lifespanOnly	3.45	3.48	5.44	4.00	2.85	3.56	2.46	3.98
GRIN2B	lifespanOnly	0.00	0.00	2.35	0.00	0.00	0.00	0.00	2.05

HNRNPA3	lifespanOnly	4.47	4.82	5.27	5.52	5.49	5.50	3.87	5.58
RETREG3	lifespanOnly	3.72	3.78	3.50	4.50	3.64	4.32	3.34	3.88
ZFPM2	weightOnly	1.09	0.96	0.00	1.99	0.82	0.37	0.26	1.90
PTCH1	weightOnly	0.00	0.00	1.86	1.76	2.08	2.02	0.00	2.43
CCDC171	weightOnly	0.29	0.10	3.65	0.98	0.11	0.32	0.00	1.55
ABCA1	weightOnly	2.60	2.39	2.80	3.12	3.16	3.51	0.85	3.36

Supplementary Notes Table SN3 *– Excerpt of the expression profiles of CpGs associated with age, lifespan, and weight in two RNA-seq samples belonging to different tissues in units of log TPM, which exemplifies expression trend differences in age-associated genes.*

Finally, we sought to determine the degree to which the average methylation score across all samples can predict average blood transcription levels for age-, weight- and lifespan-associated CpGs. We fitted a binomial linear regression model encoding presence or absence of transcription including, as response variables, average methylation, average H3K4me3 and H3K4me1 peak intensities for overlapping CpGs, and repressive chromatin state marks (H3K27me3) annotated from the human reference (**See Table SN4 below**). We found that methylation is a highly significant factor in all cases, but the predictive power of the model, measured as the odds ratio of the model's confusion matrix, was not particularly high. We did not find a significant correlation between transcription intensity and CpG methylation percentage in transcribed genes.

Variable	All CpGs	Lifespan/Weight	Age
**Methylation**	5.50E-111	9.25E-04	1.72E-34
**H3K4Me3**	9.49E-24	1.20E-01	3.18E-01
**H3K4Me1**	2.16E-67	4.01E-02	1.63E-07
**H3K27me3**	5.09E-47	2.02E-03	3.55E-01
**Odds ratio**	5.00	4.57	5.64

Supplementary Notes Table SN4 *– P-values and odds ratios of the methylation-transcription binomial models, including: all CpGs, all CpGs associated with lifespan and weight, and the top 500 CpGs associated with age.*

We observe that, while assignation of CpGs to nearby genes based on human synteny blocks (GREAT) provides a sensible framework for the enrichment and association analyses in our manuscript, not all the reported CpGs are expected to influence the transcription of their respective annotated genes. Additionally, we note that human universal chromatin state maps, as used in our manuscript, can explain up to 26.8 percent of transcription variation ($P < 10^{-200}$) in dog blood and therefore represent an acceptable proxy for general transcriptional activity in the absence of RNA-seq data.

Supplementary Note 5: Quantification of CpG distal promoter/enhancer interactions.

We queried the 5,000 bp resolution Hi-C matrix used to build the Basenji assembly (53) for distal promoter/enhancer interactions involving the mammalian methylation array CpGs. We found that CpGs associated with age, lifespan or weight, involving either *cis*- (within chromosomes) or *trans*-chromosomal interactions rarely exceeded the 10% of all CpGs associated with a given trait. Thus, while these CpGs might merit further study, we presume that a nearby gene classification of CpGs (GREAT) should capture most of the global gene enrichment trends without the need to include long-range interacting genes.

**In whole array**	**# CpGs in whole array**	**# *Cis* at least 1 contact >50000 bps**	***Trans* not calculated**
**Exon**	10895	139	#
**fiveUTR**	1149	17	#
**Intergenic_downstream**	3442	23	#
**Intergenic_upstream**	3803	20	#
**Intron**	8978	78	#
**Promoter**	1955	29	#
**threeUTR**	636	1	#
**TOTAL**	30858	307	#

**Involving age CpGs**	**~ 500 top CpGs (in age genes)**	**# *Cis* at least 1 contact >50000 bps (in age genes)**	**# *Trans* at least 1 contact (in age genes)**	**%**
**Exon**	148	3	17	13.51
**fiveUTR**	28	1	1	7.14
**Intergenic_downstream**	70	1	2	4.29
**Intergenic_upstream**	70	1	2	4.29
**Intron**	100	1	8	9.00
**Promoter**	76	0	9	11.84

**threeUTR**	3	0	0	0.00
**TOTAL**	495	7	39	9.29

**Involving lifespan/weight CpGs**	**# CpGs (in lifespan /weight genes)**	**# *Cis* at least 1 contact >50000 bps (in lifespan/weight genes)**	**# *Trans* at least 1 contact (in lifespan /weight genes)**	**%**
**Exon**	65	0	4	6.15
**fiveUTR**	9	0	2	22.22
**Intergenic_downstream**	29	0	3	10.34
**Intergenic_upstream**	33	1	0	3.03
**Intron**	108	0	7	6.48
**Promoter**	24	1	1	8.33
**threeUTR**	12	0	8	66.67
**TOTAL**	280	2	25	9.64

Supplementary Notes Table SN5 *– Number of cis and trans CpG distal interactions (further than 50,000 bps apart with scores score above the 99th quantile) for different sequence categories. Top: All CpGs in array. Middle: Top 500 Age associated CpGs. Bottom: All CpGs associated with Lifespan and Weight. Percentages are calculated adding both cis and trans interactions.*

Supplementary References

1. McInnes L, Healy J, & Melville J (2018) Umap: Uniform manifold approximation and projection for dimension reduction. *arXiv preprint arXiv:1802.03426*.
2. Friedman J, Hastie T, & Tibshirani R (2010) Regularization Paths for Generalized Linear Models via Coordinate Descent. *Journal of Statistical Software* 33(1):1-22.
3. The Kennel Club (2004) 2004 Pedigree Dog Health Survey.
4. Adams VJ, Evans KM, Sampson J, & Wood JL (2010) Methods and mortality results of a health survey of purebred dogs in the UK. *J Small Anim Pract* 51(10):512-524.
5. O'Neill DG, Church DB, McGreevy PD, Thomson PC, & Brodbelt DC (2013) Longevity and mortality of owned dogs in England. *Vet J* 198(3):638-643.
6. Leroy G, Phocas F, Hedan B, Verrier E, & Rognon X (2015) Inbreeding impact on litter size and survival in selected canine breeds. *Vet J* 203(1):74-78.
7. Lewis TW, Wiles BM, Llewellyn-Zaidi AM, Evans KM, & O'Neill DG (2018) Longevity and mortality in Kennel Club registered dog breeds in the UK in 2014. *Canine Genet Epidemiol* 5:10.
8. Proschowsky HF, Rugbjerg H, & Ersboll AK (2003) Mortality of purebred and mixed-breed dogs in Denmark. *Prev Vet Med* 58(1-2):63-74.
9. Bell L & Hesketh S (2021) Mastiff breed mortality: A study of owner experience, dog age and longevity. *Vet Anim Sci* 13:100194.
10. O'Neill DG*, et al.* (2019) West Highland White Terriers under primary veterinary care in the UK in 2016: demography, mortality and disorders. *Canine Genet Epidemiol* 6:7.
11. O'Neill DG*, et al.* (2019) Disorders of Bulldogs under primary veterinary care in the UK in 2013. *PLoS One* 14(6):e0217928.
12. O'Neill DG*, et al.* (2019) Greyhounds under general veterinary care in the UK during 2016: demography and common disorders. *Canine Genet Epidemiol* 6:4.
13. O'Neill DG, Butcher C, Church DB, Brodbelt DC, & Gough AG (2019) Miniature Schnauzers under primary veterinary care in the UK in 2013: demography, mortality and disorders. *Canine Genet Epidemiol* 6:1.
14. McGreevy PD*, et al.* (2018) Labrador retrievers under primary veterinary care in the UK: demography, mortality and disorders. *Canine Genet Epidemiol* 5:8.
15. O'Neill DG, Seah WY, Church DB, & Brodbelt DC (2017) Rottweilers under primary veterinary care in the UK: demography, mortality and disorders. *Canine Genet Epidemiol* 4:13.
16. O'Neill DG, Coulson NR, Church DB, & Brodbelt DC (2017) Demography and disorders of German Shepherd Dogs under primary veterinary care in the UK. *Canine Genet Epidemiol* 4:7.
17. Klopfenstein M, Howard J, Rossetti M, & Geissbuhler U (2016) Life expectancy and causes of death in Bernese mountain dogs in Switzerland. *BMC Vet Res* 12(1):153.
18. The American Boxer Club (2012) The American Boxer Club Health Survey Report.
19. American Chesapeake Club (2005) Chesapeake Bay Retriever Health Survey.
20. The Dalmatian Club of America (2003) The Dalmatian Club of America Health Survey Results.
21. Glickman L, Glickman N, & Thorpe R (1999) The Golden Retriever Club of America National Health Survey 1998-1999.
22. Moreland S (2008) Life Span Study of the Irish Water Spaniel in America.
23. Stivason K (2020) The Mastiff Club of America Longevity Study.
24. Toller Health Coalition (2002) Nova Scotia Duck Tolling Retriever Health Survey.
25. Erickson M*, et al.* (2009) Old English Sheepdog Club Of America 2009 Breed Health Survey Report.
26. Grayson JK (2007) West Highland White Terrier Health Survey Final Report.
27. Horvath S (2013) DNA methylation age of human tissues and cell types. *Genome biology* 14(10):R115.

28. Segre AV, *et al.* (2010) Common inherited variation in mitochondrial genes is not enriched for associations with type 2 diabetes or related glycemic traits. *PLoS genetics* 6(8).
29. Day FR, *et al.* (2015) Large-scale genomic analyses link reproductive aging to hypothalamic signaling, breast cancer susceptibility and BRCA1-mediated DNA repair. *Nature genetics* 47(11):1294-1303.
30. Day FR, *et al.* (2017) Genomic analyses identify hundreds of variants associated with age at menarche and support a role for puberty timing in cancer risk. *Nature genetics*.
31. Deelen J, *et al.* (2014) Genome-wide association meta-analysis of human longevity identifies a novel locus conferring survival beyond 90 years of age. *Human molecular genetics* 23(16):4420-4432.
32. Fritsche LG, *et al.* (2013) Seven new loci associated with age-related macular degeneration. *Nature genetics* 45(4):433-439, 439e431-432.
33. Genetic Modifiers of Huntington's Disease C (2015) Identification of Genetic Factors that Modify Clinical Onset of Huntington's Disease. *Cell* 162(3):516-526.
34. Lambert JC, *et al.* (2013) Meta-analysis of 74,046 individuals identifies 11 new susceptibility loci for Alzheimer's disease. *Nature genetics* 45(12):1452-1458.
35. Lango Allen H, *et al.* (2010) Hundreds of variants clustered in genomic loci and biological pathways affect human height. *Nature* 467(7317):832-838.
36. Liu JZ, *et al.* (2015) Association analyses identify 38 susceptibility loci for inflammatory bowel disease and highlight shared genetic risk across populations. *Nature genetics* 47(9):979-986.
37. Locke AE, *et al.* (2015) Genetic studies of body mass index yield new insights for obesity biology. *Nature* 518(7538):197-206.
38. Major Depressive Disorder Working Group of the Psychiatric GC, *et al.* (2013) A mega-analysis of genome-wide association studies for major depressive disorder. *Molecular psychiatry* 18(4):497-511.
39. Manning AK, *et al.* (2012) A genome-wide approach accounting for body mass index identifies genetic variants influencing fasting glycemic traits and insulin resistance. *Nature genetics* 44(6):659-669.
40. Morris AP, *et al.* (2012) Large-scale association analysis provides insights into the genetic architecture and pathophysiology of type 2 diabetes. *Nature genetics* 44(9):981-990.
41. Neale BM, *et al.* (2010) Meta-analysis of genome-wide association studies of attention-deficit/hyperactivity disorder. *Journal of the American Academy of Child and Adolescent Psychiatry* 49(9):884-897.
42. Okbay A, *et al.* (2016) Genome-wide association study identifies 74 loci associated with educational attainment. *Nature* 533(7604):539-542.
43. Psychiatric GCBDWG (2011) Large-scale genome-wide association analysis of bipolar disorder identifies a new susceptibility locus near ODZ4. *Nature genetics* 43(10):977-983.
44. Schizophrenia Working Group of the Psychiatric Genomics C (2014) Biological insights from 108 schizophrenia-associated genetic loci. *Nature* 511(7510):421-427.
45. Shungin D, *et al.* (2015) New genetic loci link adipose and insulin biology to body fat distribution. *Nature* 518(7538):187-196.
46. Simon-Sanchez J, *et al.* (2009) Genome-wide association study reveals genetic risk underlying Parkinson's disease. *Nature genetics* 41(12):1308-1312.
47. McCartney DL, *et al.* (2021) Genome-wide association studies identify 137 genetic loci for DNA methylation biomarkers of aging. *Genome biology* 22(1):194.
48. Elsworth B, *et al.* (2020) The MRC IEU OpenGWAS data infrastructure. *bioRxiv*:2020.2008.2010.244293.
49. Lyon M, *et al.* (2020) The variant call format provides efficient and robust storage of GWAS summary statistics. *bioRxiv*:2020.2005.2029.115824.
50. Hemani G, *et al.* (2018) The MR-Base platform supports systematic causal inference across the human phenome. *eLife* 7.

51. Broad Institute Broadinstitute/GTEX-Pipeline: Gtex & topmed Data Production and Analysis Pipelines. *GitHub*. Available at: https://github.com/broadinstitute/gtex-pipeline [Accessed March 9, 2022].
52. Evans JM, *et al*. (2021) Multi-omics approach identifies germline regulatory variants associated with hematopoietic malignancies in retriever dog breeds. *PLOS Genetics* 17(5).
53. Edwards RJ, *et al*. (2021) Chromosome-length genome assembly and structural variations of the primal basenji dog (canis lupus familiaris) genome. *BMC Genomics* 22(1).
